# Integrative analysis of CRISPR screening data uncovers new opportunities for optimizing cancer immunotherapy

**DOI:** 10.1186/s12943-021-01462-z

**Published:** 2022-01-02

**Authors:** Yan Li, Chen Yang, Zhicheng Liu, Shangce Du, Susan Can, Hailin Zhang, Linmeng Zhang, Xiaowen Huang, Zhenyu Xiao, Xiaobo Li, Jingyuan Fang, Wenxin Qin, Chong Sun, Cun Wang, Jun Chen, Huimin Chen

**Affiliations:** 1 State Key Laboratory for Oncogenes and Related Genes; Key Laboratory of Gastroenterology & Hepatology, Ministry of Health; Division of Gastroenterology and Hepatology; Shanghai Institute of Digestive Disease; Renji Hospital, Shanghai Jiao Tong University School of Medicine, 145 Middle Shandong Road, Shanghai, 200001 China; 2 Zhongshan School of Medicine, Sun Yat-sen University, Guangzhou, 510080 China; 3 State Key Laboratory of Oncogenes and Related Genes, Shanghai Cancer Institute, Renji Hospital, Shanghai Jiao Tong University School of Medicine, Shanghai, 200001 China; 4 Hepatic Surgery Center, Tongji Hospital, Tongji Medical College, Huazhong University of Science and Technology, Wuhan, 430030 China; 5 Immune Regulation in Cancer Group, German Cancer Research Center (DKFZ), Heidelberg, 69120 Germany; 6 Guangdong Provincial Key Laboratory of Malignant Tumor Epigenetics and Gene Regulation, Sun Yat-Sen Memorial Hospital, Sun Yat-Sen University, Guangzhou, 510120 China; 7 Key Laboratory of Tropical Disease Control of the Ministry of Education, Sun Yat-sen University, Guangzhou, 510080 China; 8 Guangdong Engineering & Technology Research Center for Disease-Model Animals, Laboratory Animal Center, Zhongshan School of Medicine, Sun Yat-sen University, Guangzhou, 510080 China; 9 Center for Precision Medicine, Sun Yat-sen University, Guangzhou, 510080 China

**Keywords:** CRISPR screen, Tumor immunity, MON2, Immune checkpoint blockade, Drug repurposing, Connectivity map

## Abstract

**Background:**

In recent years, the application of functional genetic immuno-oncology screens has showcased the striking ability to identify potential regulators engaged in tumor-immune interactions. Although these screens have yielded substantial data, few studies have attempted to systematically aggregate and analyze them.

**Methods:**

In this study, a comprehensive data collection of tumor immunity-associated functional screens was performed. Large-scale genomic data sets were exploited to conduct integrative analyses.

**Results:**

We identified 105 regulator genes that could mediate resistance or sensitivity to immune cell-induced tumor elimination. Further analysis identified *MON2* as a novel immune-oncology target with considerable therapeutic potential. In addition, based on the 105 genes, a signature named CTIS (CRISPR screening-based tumor-intrinsic immune score) for predicting response to immune checkpoint blockade (ICB) and several immunomodulatory agents with the potential to augment the efficacy of ICB were also determined.

**Conclusion:**

Overall, our findings provide insights into immune oncology and open up novel opportunities for improving the efficacy of current immunotherapy agents.

**Supplementary Information:**

The online version contains supplementary material available at 10.1186/s12943-021-01462-z.

## Background

The advent of immune checkpoint blockade (ICB) therapies, such as blockades of programmed cell death 1 (PD-1), programmed cell death-ligand 1 (PD-L1), or cytotoxic T-lymphocyte antigen-4 (CTLA-4), have revolutionized the treatment modalities of many cancer types [1]. Currently, several agents, including ipilimumab (anti-CTLA-4) [2], pembrolizumab and nivolumab (anti-PD-1) [3, 4], and atezolizumab (anti-PD-L1) [5], have been approved by the Food and Drug Administration (FDA) for clinical use. Besides, similar immune-oncology agents targeting LAG3 [6], TIM3 [7] and TIGIT [8] are being investigated in ongoing preclinical and clinical studies. Despite the significant advances made in cancer immunotherapy, there still exist many challenges in this field, such as low response rates and acquired resistance [9]. Accordingly, more efforts are required to elucidate the mechanisms underlying sensitivity or resistance to antitumor immune response and develop more efficient immunotherapeutic strategies.

Recently, clustered regularly interspaced short palindromic repeats (CRISPR)/Cas9 technology-based functional screens have been successfully applied to dissect potential factors regulating tumor immunity, providing a new paradigm for target discovery in a high-throughput manner [10–13]. Both loss-of-function and gain-of-function screens can be realized using CRISPR/Cas9-based systems [14]. Nonetheless, due to the relative complexity of the gain-of-function screening technology, loss-of-function screening is currently the most commonly adopted approach. Depending on the experimental model, screens can also be classified into two types, namely in vitro and in vivo screens [15]. Tumor/immune co-culture systems are the basis of in vitro screens, which can easily mimic the interaction between tumor and immune cells in in vitro cultures [15]. Compared to in vitro models, in vivo models benefit from a preserved immune system and an intact tissue microenvironment and are thus more likely to identify clinically significant targets [15].

To date, immuno-oncology screens have yielded a substantial amount of high-throughput data. However, no systematic analysis has been carried out to integrate these data to the best of our knowledge. The present study begins to fill this gap by performing a comprehensive literature retrieval for immune-oncology screens in publicly available databases. Results from these screens were then integrated to identify potential regulators engaged in tumor immunity. Novel immuno-oncology targets with therapeutic implications and transcriptome signatures for predicting response to cancer immunotherapy were determined by leveraging screening-derived results and multi-omics data from TCGA Pan-cancer cohort and ICB-treated datasets. More importantly, we identified potential immunomodulatory agents that exhibited a synergistic effect with ICBs via an improved signature matching approach. Overall, our findings may provide a treasure trove for future studies on antitumor immunity and hold the potential to improve the efficacy of current immunotherapy regimens.

## Materials and methods

### CRISPR screening analysis

A total of 22 screens from 11 different studies were included in this study. Of these, 17 screens were focused on investigating the regulators of immune cell-mediated killing, while the other five screens introduced the ICB treatment into their experiments which were used to identify regulators mediating response to cancer immunotherapies. Data of these screens were achieved from the supplementary files of corresponding publications. Some included studies only provided processed data, while some also provided raw count data. For those without raw data, processed data was used directly for subsequent analyses; for those providing raw data, MAGeCK pipeline (v0.5.9) with default parameters was utilized to identify the significantly altered genes and single guide RNAs (sgRNAs) [16]. Enriched genes were defined as those with positive adj. *P* < 0.05 and log-fold change > 0 and depleted genes were defined as those with negative adj. *P* < 0.05 and log-fold change < 0. For those screens using mouse models, the resultant mouse genes were mapped to orthologous human genes using *biomaRt* package, and genes without known homologous relationships were excluded from subsequent analysis.

### Definition of gene functional status

For screen-derived genes and tumor suppressor genes (TSGs), functionally relevant events were considered as gene inactivation. The definition of inactivation events required support from multi-omics data. Briefly, the mutation data were first preprocessed, and deleterious mutations were defined as loss-of-function mutations (including frameshift, stopgain, startloss and stoploss) or missense mutations predicted as possibly or probably damaging (probability score > 0.5) by PolyPhen2 [17]. Samples with inactivation events were then defined as samples presenting deleterious mutations, deep deletions (GISTIC value = − 2), or scaled-expression ≤ − 2. For oncogenes (OGs), functionally relevant events were considered as gene activation. Considering that the computational prediction of gain-of-function mutations is more challenged than loss-of-function mutations with relatively low accuracy, mutation data was excluded from the definition of activation events [18]. Accordingly, Samples with activation events were then defined as samples presenting high-level amplification (GISTIC value = 2) or scaled-expression ≥2.

### Statistical analysis

Statistical analysis and graphical visualization were all performed in R, version 3.6.0 (https://cran.r-project.org/). Depending on the data type, comparison of continuous variables in two or more than two groups was performed using either parametric test (Student’s t-test or analysis of variance) or nonparametric test (Wilcoxon rank-sum test or Kruskal-Wallis test), and correlation between two continuous variables was measured by either Pearson correlation or Spearman rank correlation. Contingency table variables were analyzed by Fisher’s exact tests. Survival analysis was performed based on Kaplan-Meier methods and the statistical significance of differences was determined using the log-rank (Mantel-Cox) test. For those analyses with more than 20 comparisons, multiple testing correction was conducted with FDR method. The statistical details including the statistical test used for each dataset are indicated in the figure legends. Unless otherwise stated, a *p*-value of 0.05 was considered as being statistically significant. Additional detailed methods can be found in the Supplemental Materials and Methods.

## Results

### Summary of included CRISPR screens

**Fig. 1:**
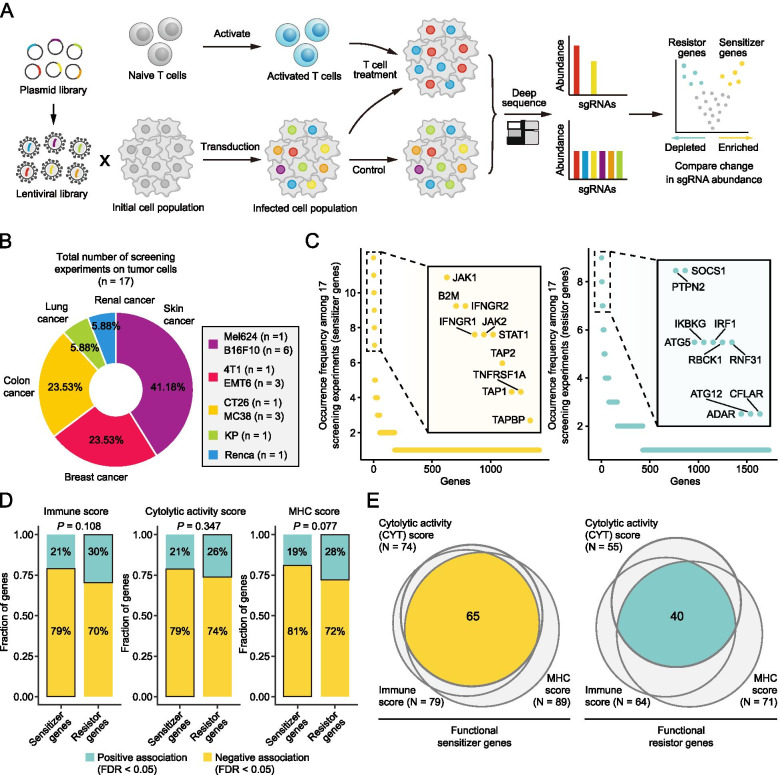
Identification of regulators involved in antitumor immune response by integrative analyses of immune-oncology screening data. **A** Schematic illustration of the process of pooled CRISPR/Cas9 knockout screens using tumor/immune co-culture systems. This screening approach was adopted by most of the included experiments. **B** Distribution of screens across different cancer types. **C** Common sensitizer genes (left) and resistor genes (right) identified by different screens. The top 10 genes were labeled in each plot. **D** Association between the functional status of sensitizers/resistors and the abundance of immune/cytolytic activity/MHC scores. Statistical significance of associations was determined using regression analysis, adjusting for cancer type. Comparison between sensitizers and resistors was conducted using Fisher’s exact tests. **E** Intersections of the resultant genes from three different filtering approaches. The results of sensitizers (left) and resistors (right) were displayed separately

In this study, a total of 17 CRISPR screens on the regulators of immune-mediated tumor elimination were included (the other five ICB-treated screens were described in the following section) (Table S1). To improve comprehension, a schematic diagram describing the process of CRISPR screen in a tumor/immune co-culture system is shown in Fig. 1A. Five cancers were selected as research objects, among which skin cancer (41.18%) was the most commonly studied cancer model, followed by breast cancer (23.53%) and colon cancer (23.53%) (Fig. 1B). The distribution of screens among different screening types, library types, organisms, and algorithms was also analyzed. We found that most of the included screens were carried out under in vitro conditions (76.47%), using a genome-scale library (88.24%) and mouse-derived cell line models (94.12%). Moreover, DrugZ (47.06%) and MAGeCK (35.29%) were the two most adopted algorithms for data analysis (Fig. S1). Notably, the included screens all used CRISPR knockout (CRISPRko) libraries, which were designed to investigate the relationship between the loss-of-function of genes and corresponding phenotypes.

### Determination of antitumor immunity-related regulators

Based on data from these screens, we sought to find out potential regulator genes involved in antitumor immunity. Each screen could identify a plethora of enriched or depleted single guide RNAs (sgRNAs). Considering that no gain-of-function screens were included in this study, results from all the screens here could be interpreted in the same manner. Briefly, the knockout of genes that exert positive effects on anti-tumor immune response (such as genes associated with antigen presentation) resulted in resistance of corresponding tumor cells to T-cell mediated killing, thus enriching their sgRNAs (these genes were named as sensitizer genes hereafter). Conversely, the knockout of genes that promote tumor immune escape (such as *CD274*) can enhance the sensitivity of tumor cells to T cell-mediated killing, leading to the depletion of sgRNAs (these genes were named as resistor genes hereafter). The sensitizer and resistor genes in each screen were identified respectively. A tumor type-dependent result can be found in Table S2. All the results from different screens were then integrated and only those genes presented across two or more screens were selected for further analyses (Fig. 1C). This step yielded a preliminary list of 181 sensitizers and 427 resistors.

Given that the overall accuracy of screening results remained far from perfect, we intended to further narrow down this gene list to obtain more reliable candidate regulators. To this end, three well-proven tumor immunity-related gene signatures, namely ESTIMATE-based immune signature [19], cytolytic activity (CYT) signature [20], and MHC signature [21], were leveraged to discern sensitizer and resistor genes with potential functional significance. Specifically, we first calculated the corresponding scores of these three signatures based on the expression profiles from the TCGA Pan-Cancer cohort [22]. The functional status (a dichotomous variable, 0 = non-inactivation; 1 = inactivation) of each candidate sensitizer/resistor gene in each TCGA sample was determined using multi-omics TCGA data, which included expression, mutation, and copy number variation (CNV) data (see Materials and methods section). Given that all the candidate regulators were derived from the results of CRISPRko screens, we defined loss-of-function (inactivating) status as the main functional event. Then, the associations between the functional status of each sensitizer/resistor gene and the abundance of each signature could be determined by adopting a regression-based approach. After controlling for cancer type and adjusting for the multiple testing, associations with adj. *P* < 0.05 were deemed statistically significant. It should be noted that higher scores obtained for the three signatures indicated an enhanced anti-tumor immune response [23]. Therefore, according to the definition of sensitizer/resistor genes, sensitizers should be negatively associated with these signatures (lower scores upon inactivation), while resistors should be positively associated (higher scores upon inactivation). As indicated by the results, resistors exhibited an incline for a higher proportion of positive associations, albeit not significant, which could prove the rationality of this filtering approach to some extent (Fig. 1D). Functional sensitizer genes were defined as genes with significant negative associations (adj. *P* < 0.05 and coefficient < 0) with all three signatures (*n* = 65), while functional resistor genes were defined as genes with significant positive associations (adj. *P* < 0.05 and coefficient > 0) with all three signatures (*n* = 40) (Fig. 1E, Table S3).

### Functional analysis of sensitizers and resistors

**Fig. 2:**
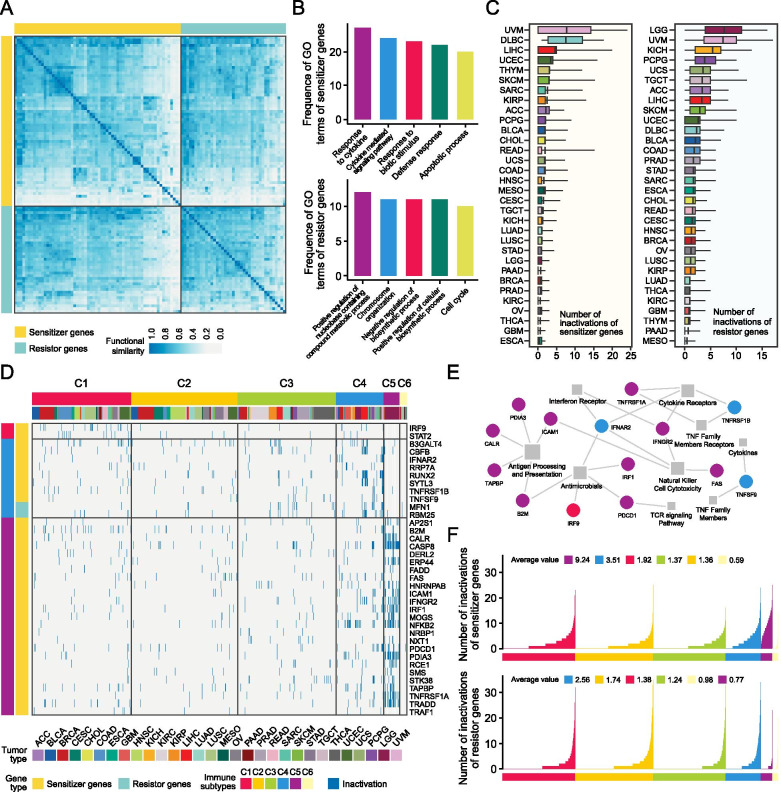
The overall characterization of sensitizer and resistor genes. **A** Similarity matrix representing the Gene Ontology (GO)-based functional similarity (FS) scores between each pair of sensitizers/resistors. **B** GO functional annotation of sensitizer genes (upper) and resistor genes (lower) based on the occurrence frequency. Only the top five terms were presented. **C** Distribution of the inactivation event numbers of sensitizers (left) and resistors (right) across different TCGA cancer types. **D** Heatmap showing the inactivation events of subtype-specific sensitizer/resistor genes across six immune subtypes. Subtype-specific genes were determined using logistic regression. Only statistically significant genes were presented. **E** Functional annotation of subtype-specific sensitizer/resistor genes using information from ImmPort (immport.org). **F** Distribution of the inactivation event numbers of sensitizers (upper) and resistors (lower) across different immune subtypes

We conducted gene ontology (GO)-based functional similarity (FS) and annotation analysis to determine the functional characteristics of these sensitizer and resistor genes. The FS scores of each gene pair across all 105 genes were first computed and visualized on a heatmap (Fig. 2A). Besides, the distribution of FS scores of each sensitizer and resistor gene was also analyzed (Fig. S2A). Conceptually, a gene that had higher similarity with others was more likely to possess a central or significant role. In this regard, some critical transcription factor genes involved in tumor immunity, such as *STAT1* and *STAT2*, were observed to have relatively high similarities with other genes. Intriguingly, the comparison between sensitizer and resistor genes suggested that resistors had significantly higher internal similarity (*P* = 0.0001, Fig. S2B). To characterize the biological processes related to these sensitizers and resistors, we next performed functional annotation based on the occurrence frequency of GO-BP terms from the Molecular Signatures Database (MSigDB) [24]. The results suggested that sensitizer genes tended to engage in immune-related processes, while resistor genes were mostly involved in some metabolic and biosynthetic processes (Fig. 2B, Table S4).

### Dysregulation patterns of sensitizers and resistors

We also sought to explore the dysregulated expression patterns of these genes across different cancer types and immune subtypes. First, we focused on investigating the expression pattern of genes between tumor tissues and adjacent tissues. Differential expression analysis was conducted on 105 sensitizer and resistor genes across cancers in both tumor and normal tissues (*n* > 2). The results suggested that the differential patterns of the two gene types were inconsistent across different cancer types. Overall, in 57.9% of all cancer types, a numerically higher proportion of up-regulated resistor genes could be observed (Fig. S3). Next, the distribution of the inactivation events of sensitizer and resistor genes across different cancer types was analyzed (Fig. 2C). A more objective relationship was observed between resistors and sensitizers in each cancer type by calculating the normalized resistor-sensitizer ratio (Fig. S4A).

In addition, we also delineated the relationship between the dichotomous functional status of these genes and multi-class immune subtypes from a previous publication (C1-C6) [25]. To identify subtype-specific sensitizers or resistors, a logistic regression-based approach was adopted, and genes with adj. *P* < 0.05 and log2 odds ratio (OR) > 1.5 in certain immune subtypes were defined as the specific genes of this subtype. The analysis yielded a total of 37 subtype-specific genes (35 out of 37 were sensitizers), including two C1-specific genes, 10 C4-specific genes, and 25 C5-specific genes (Fig. 2D). The functions of these genes were then annotated using GO information from ImmPort (immport.org) [26]. Interestingly, we observed that the inactivation events of multiple C5-specific genes were related to the process of antigen processing and presentation (Fig. 2E). Then, the distribution of the inactivation events of sensitizers and resistors across six immune subtypes was analyzed (Fig. 2F), and the corresponding normalized sensitizer-resistor ratios were also calculated (Fig. S4B). Among the six subtypes, C5 had the highest average number of inactivation events of sensitizers (*n* = 9.24) as well as the highest normalized sensitizer-resistor ratio (r = 2.89), which was consistent with its property as an immunologically quiet subtype [25].

### The functional characterization of sensitizers and resistors across cancers

**Fig. 3:**
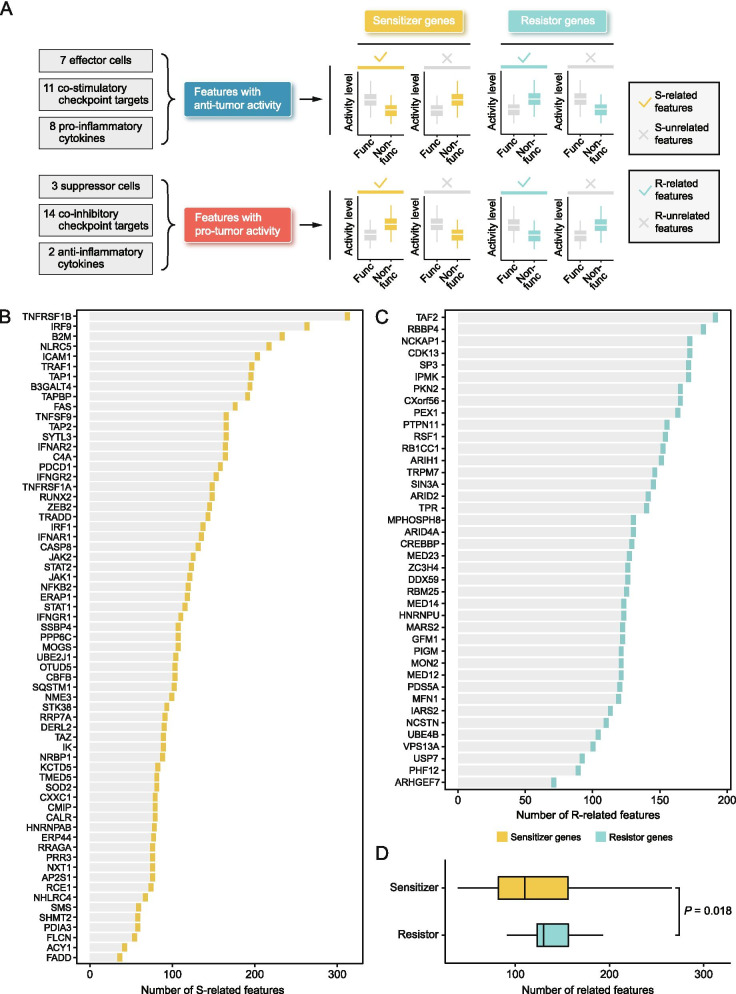
Functional determination of sensitizers and resistors. **A** Visualization of the definition of S−/R-related features. **B** The total number of S-related features across all the cancer types of sensitizer genes. **C** The total number of R-related features across all the cancer types of resistor genes. **D** Comparison of the numbers of related features between sensitizers and resistors. Statistical significance of difference was determined using Wilcoxon rank-sum test

Theoretically, the loss of sensitizer genes (such as *B2M*) should enable tumors to resist immune attack, while the loss of resistor genes (such as *CD274*) could augment the cytotoxic effects of immune cells on tumors. However, the actual functions of these genes may vary across different cancer types. To characterize the actual functions of sensitizers and resistors within each cancer type, we designed a computational approach (Fig. 3A). Briefly, we first manually curated a list of immune-related features with anti−/pro-tumor activity, including immune cells, immune checkpoint genes, and inflammatory cytokines (Table S5). Then, the associations between the dichotomous status of sensitizers/resistors and the abundance of immune-related features were calculated using a regression-based approach. Sensitizer-related (S-related) features were defined as anti-tumor features with negative association (lower activity upon inactivation) or pro-tumor features with a positive association (higher activity upon inactivation) with sensitizer genes. Resistor-related (R-related) features were defined in an analogous way. Accordingly, the number of S−/R-related features for each sensitizer or resistor genes in each cancer type could be determined (Fig. S5A-B). Conceptually, a sensitizer/resistor with greater S−/R-related feature number is more likely to have a crucial role in anti-tumor immunity. We also calculated the total S−/R-related feature number across different cancers of sensitizers and resistors, respectively (Fig. 3B, C). Notably, it could be observed that some well-recognized immune regulators, such as *B2M* and *TAP1*, were relatively top-ranked. Besides, we also found that resistor genes have more related features than sensitizer genes (Fig. 3D).

### Identification of MON2 as novel immuno-oncology target

**Fig. 4:**
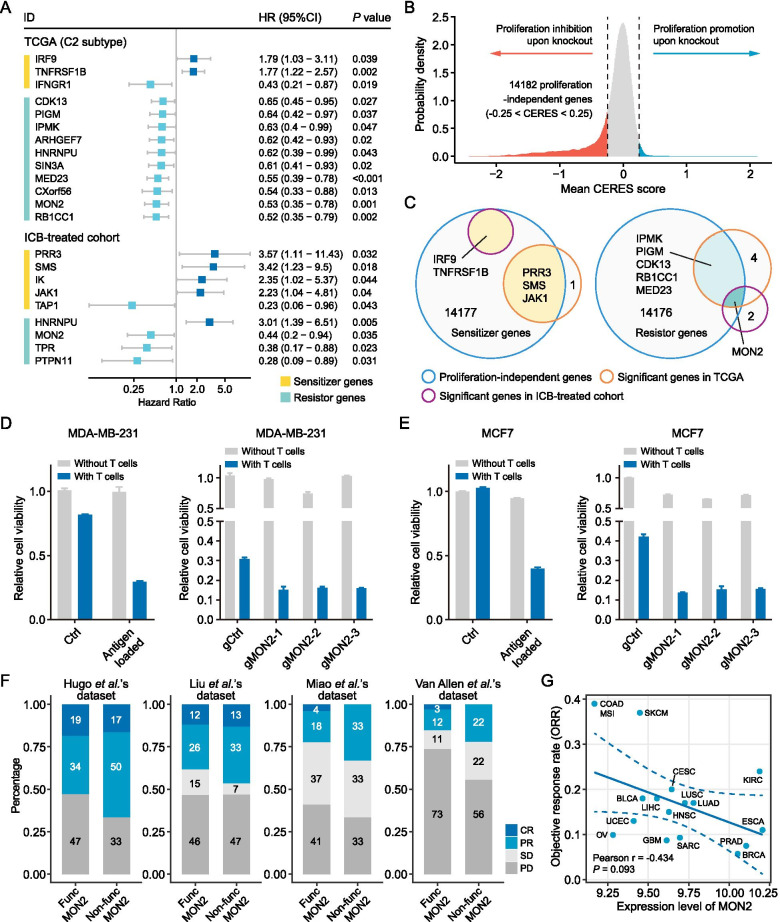
Determination of *MON2* as novel immuno-oncology target. **A** Presentation of sensitizers and resistors with prognostic significance in TCGA (C2 subtype) and ICB-treated cohorts. The hazard ratio (HR) and corresponding 95% confidence interval (CI) were estimated using a Cox regression model, adjusting for age and cancer type. **B** Determination of proliferation-independent genes according to the CERES scores from CRISPR knockout screens across hundreds of cancer cell lines. **C** Intersections between proliferation-independent genes and sensitizer (left) and resistor (right) genes with prognostic significance in both TCGA and ICB-treated cohort. **D** Coculture assay of MDA-MB-231 cells and antigen-specific T cells. MDA-MB-231 was loaded with Mart1 epitope by lentiviral transduction and cultured in the absence or presence of Mart-1- specific T cells (left panel). MDA-MB-231 cells that express Mart-1-epitope were transduced with Cas9 and then three independent gRNAs targeting MON2. A non-targeting gRNA served as a control. The cells were cultured with or without Mart-1-specific T cells for 24 h (right panel). **E** Coculture assay of MCF7 cells and antigen-specific T cells. **F** Association between the functional status of *MON2* and clinical response to immunotherapy (CR, complete response; PR, partial response; SD, stable disease; PD, progressive disease) in four ICB-treated datasets. **G** Pearson correlation between *MON2* expression and objective response rate (ORR) for ICB. Only non-zero data was included

To investigate whether the functional status of sensitizers and resistors was associated with the survival outcome of cancer patients, we conducted Cox proportional hazards regression analysis for all the 105 sensitizer and resistor genes, controlling for confounding factors including cancer type and age. The immune subtype of C2 was found to have an immune-inflamed phenotype, and thus there might exist more interactions between cancer cells and cytotoxic immune cells in tumors of this subtype [25]. Therefore, for the C2 subtype, the impact of the functional status of sensitizers and resistors on prognosis was more likely to be mediated by antitumor immunity-associated mechanisms. Accordingly, only 2591 cases in the C2 subtype were utilized to conduct this analysis, which yielded 3 and 10 significant prognostic sensitizer and resistor genes, respectively (Fig. 4A). Besides, we explored the prognostic associations of sensitizers and resistors in ICB-treated datasets. A total of 10 datasets from eight different studies were collected, which were then integrated into a metadata set (Table S6). The dichotomous functional status of sensitizer and resistor genes in this set was defined similarly to the TCGA Pan-Cancer cohort. Survival analysis in the metadata set identified nine significant prognostic genes, including five sensitizers and four resistors (Fig. 4A). Interestingly, it could be observed that most of the sensitizers (75%) served as unfavorable prognostic factors (hazard ratio > 1 upon inactivation), while most of the resistors (93%) were associated with favorable prognosis (hazard ratio < 1 upon inactivation), which was in accordance with their own properties.

Considering that the loss of a certain gene in itself may affect the survival of cancer cells without the involvement of antitumor immune response, we performed an additional filtering procedure using gene dependency data from the Cancer Dependency Map (DepMap) portal (depmap.org) to avoid potential bias introduced by essential genes [27]. The average CERES scores across 739 cancer cell lines were calculated, and genes with CERES scores ranging from − 0.25 to 0.25 were considered tumor proliferation-independent genes (*n* = 14,182) (Fig. 4B). By intersecting the above results, a resistor named *MON2* was identified as the only proliferation-independent gene, which had significant prognostic associations both in the TCGA C2 and combined ICB-treated cohorts (Fig. 4C).

### Association of MON2 with antitumor immunity

Systematic analyses were conducted to characterize the roles of *MON2* in antitumor immunity. *MON2* was identified as a significant resistor gene by two of the 17 screens (Fig. S6A). Given that one of the screens was carried out in cells derived from mouse breast cancer, we validated our finding in two human breast tumor cell lines, MDA-MB-231 and MCF7. *MON2*-deficient tumor cells were generated by CRISPR/Cas9. The knockout efficiency was determined by the Tracking of Indels by Decomposition (TIDE) [28] (Fig. S6B). The resulting cells were loaded with a defined antigen and co-cultured with TCR-transduced T cells that recognize the antigen-MHC class I complex. We observed that both MDA-MB-231 and MCF7 cells with *MON2* deficiency were more sensitive to the killing effect of T cells than MON2-proficient cells, consistent with the results from the CRISPR screen in mouse cells (Fig. 4D, E).

We further interrogated the relationship between *MON2* and the three above scores, including immune, CYT, and MHC scores. *MON2* was most significantly associated with MHC scores (Fig. S7A). Since MHC scores were used to measure the antigen presentation ability of tumor cells, we hypothesized that *MON2* might be more relevant to tumor cell-intrinsic immune-associated factors. To validate this hypothesis, the associations between *MON2* and the other three tumor cell-intrinsic factors, including mutational load, single nucleotide variant (SNV)-based neoantigen load, and insertion and deletion (indel)-based neoantigen load, were tested. As expected, a high association was found between *MON2* and all three factors (Fig. S7B). These analyses collectively showed that the loss of *MON2* was closely related to enhanced tumor immunogenicity.

Based on the response data from ICB-treated datasets, we next set out to evaluate whether a direct correlation was present between *MON2* and response to ICBs. Unsurprisingly, the loss of *MON2* was associated with a more favorable response to ICBs in multiple datasets, albeit not very remarkable (Fig. 4F). As a complementary investigation, the relationship between the expression of *MON2* and the objective response rate (ORR) of anti-PD-1/PD-L1 therapy was investigated as well [29]. A marginally significant negative correlation between *MON2* expression and ORR was obtained (*P* = 0.093), which could corroborate the above conclusion to some extent (Fig. 4G). Given that *MON2* expression could be a potential factor affecting immunotherapy response, we delineated the distribution of *MON2* expression across different cancer types (Fig. S7C) and immune subtypes (Fig. S7D). Notably, it could be observed that *MON2* exhibited the highest expression level in the C5 subtype (immunologically quiet). Furthermore, the crucial role of microsatellite instability (MSI) status in the field of cancer immunotherapy prompted us to examine the association between *MON2* expression and MSI status [30]. Three cancer types in TCGA, including COAD, STAD, and UCEC, were selected for conducting this analysis with a large sample size of MSI-high (MSI-H) tumors [31]. MSI-low (MSI-L)/microsatellite-stable (MSS) tumors showed a significantly higher expression of *MON2* in COAD, STAD, and the combined cohorts (Fig. S8A, B, D), except for UCEC (Fig. S8C). Correlation analysis between MSI events and *MON2* expression was also conducted, and a significant negative correlation was obtained, as expected (*P* = 0.004) (Fig. S8E). Of note, MSI-L/MSS tumors as well as tumors in C5 subtypes were considered to be resistant to currently approved ICBs [25, 32, 33]. Collectively, inhibition of *MON2* might lead to the augmentation of anti-tumor immunity, which might open new possibilities for treating tumors with high *MON2* activity.

### Construction of CTIS for predicting immunotherapy response

Despite recent advances, predicting response to immunotherapy remains challenging and requires further investigations. As our findings suggested important roles of sensitizer and resistor genes in immune cell-mediated tumor killing, we postulated that it might be clinically meaningful to construct a predictive signature based on these genes.

**Fig. 5:**
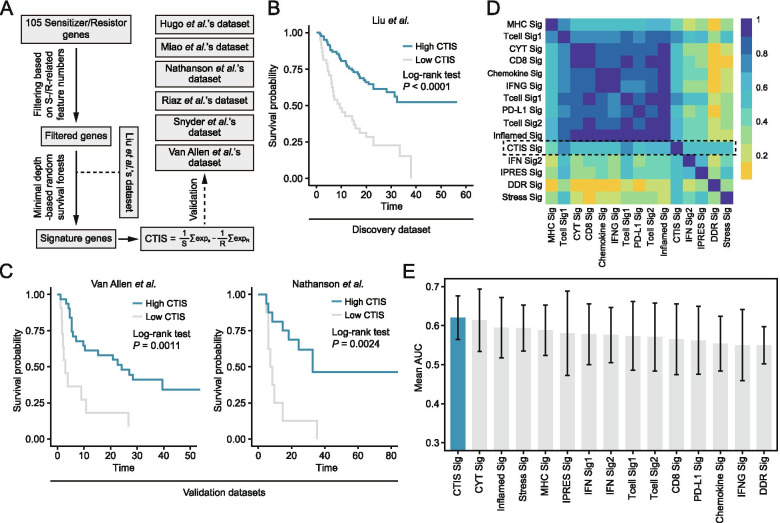
Construction of CTIS for predicting immunotherapeutic response. **A** Workflow of the construction and evaluation of CTIS. **B** Survival significance of CTIS in discovery dataset. **C** Survival of CTIS in two validation datasets. **D** Similarity comparison between the CTIS signature and other 14 published signatures. **E** Comparison of mean AUC values across all the pretreatment datasets between CTIS and other published signatures

The detailed process of signature construction was displayed in Fig. 5A. Only pretreatment samples were included in this step. A preliminary filtering was first performed to exclude genes with less than 100 S−/R-related features (across all the cancer types). As a result, 39 sensitizers and 37 resistors were retained after the filtration. Then, based on Liu et al.’s dataset which has the largest pretreatment sample size, minimal depth (MD)-based random survival forest (RSF) analysis was conducted to further narrow down the gene list [34]. The RSF analysis was repeated 1000 times and six genes that led to a largest concordance index (C-index) value was considered as the final candidates. These genes consisted of four sensitizers (JAK1, NFKB2, PPP6C and TNFRSF1B) and two resistors (PIGM and TPR). A CRISPR screening-based tumor-intrinsic immune score (CTIS) was then defined as the difference between the mean expression of four sensitizers and two resistors and a higher CTIS indicated a stronger anti-tumor immune response. In the discovery dataset (Liu et al.’s dataset), CTIS exhibited a significant prognostic value (*P* < 0.0001); higher CTIS was related to a better survival outcome (Fig. 5B). We also investigated its performance in several validation datasets. Among them, we found CTIS was significantly associated with prognosis in two datasets and patients with higher CTIS consistently had an improved overall survival (Fig. 5C).

After that, we comprehensively compared the CTIS signature with other 14 published signatures (details in Supplemental Materials and Methods). The association between these signatures was first investigated using the expression data of ICB-treated datasets. It could be observed that many signatures, such as CD8, CYT, IFNG, PD-L1, and inflamed signatures, were highly correlated with each other (Fig. 5D). In contrast, the CTIS signature presented relatively moderate associations with other signatures, suggesting it has a complementary rather than an alternative role. Then, comparison of the performance for predicting response to ICB treatment between CTIS and other signatures was performed (Fig. S9). Although CTIS was not the best biomarker across all the datasets, it outperformed other signatures in the condition of comparing the overall performance, with a mean AUC value of 0.620 (Fig. 5E).

We further calculated the CTIS of treatment-naïve tumors from TCGA Pan-Cancer cohort to characterize the distribution of CTIS across different cancer types (Fig. S10A) and immune subtypes (Fig. S10B). Among the six immune subtypes, C5 had the lowest CTIS scores, consistent with its property. Although higher CTIS indicated a better prognosis in ICB-treated datasets, in treatment-naïve TCGA tumors, CTIS conferred a dual prognostic impact depending on the cancer type (Fig. S10C). In addition, relationships among mutation and neoantigen load and CTIS were also assessed. However, no remarkable results were observed (Fig. S10D).

### Determination of potential OGs/TSGs regulatory network

**Fig. 6:**
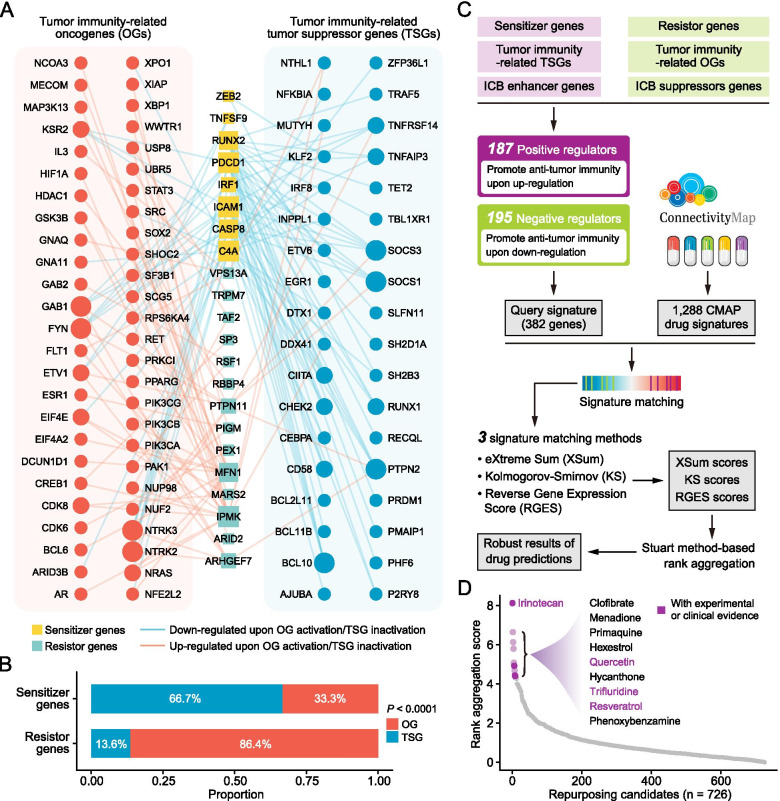
Identification of potential immunomodulatory agents for potentiating the efficacy of immunotherapies. **A** Bipartite network showing the interactions between sensitizer/resistor genes and OGs/TSGs. Node size is proportional to the interaction degree; a node with larger size represents that it has more interactions with other nodes. **B** The proportion of interactions with OGs and TSGs in sensitizers and resistors. Statistical significance was determined using Fisher’s exact tests. **C** Computational workflow of the inference of potential immunomodulatory agents using the signature matching approach. **D** Result of drug prediction. The top 10 drugs were labeled in the plot

It has been widely reported that some oncogenes (OGs) or tumor suppressor genes (TSGs) may be involved in the regulation of tumor immunity [35]. For example, activation of *MYC* can induce the transcription of both CD47 and PD-L1 and thereby suppress the anti-tumor immune response [36]. Accordingly, we considered that it was reasonable to build an OGs/TSGs regulatory network for sensitizers and resistors, which could uncover potential mechanisms underlying the dysregulation of sensitizers and resistors. The list of OGs and TSGs was obtained from OncoKB (oncokb.org) [37]. For OGs, functionally relevant events were considered as activation (0 = non-activation; 1 = activation). As for TSGs, functionally relevant events were considered as inactivation (0 = non-inactivation; 1 = inactivation). We hypothesized that associations existed between sensitizers and OGs/TSGs when the expression of sensitizers was significantly down-regulated upon OG activation/TSG inactivation (adj. *P* < 0.05 and log-fold change < − 0.25). Correspondingly, an association between resistors and OGs/TSGs was found when resistors’ expression was significantly up-regulated upon OG activation/TSG inactivation (adj. *P* < 0.05 and log-fold change > 0.25). We identified a total of 159 significant associations between sensitizers/resistors and OGs/TSGs (Fig. 6A). Interestingly, we found that sensitizer genes tended to have more associations with TSGs (66.7%), while resistor genes exhibited considerably more associations with OGs (86.4%) (Fig. 6B). OGs/TSGs with significant associations were defined as tumor immunity-related OGs/TSGs (Table S7).

### Prediction of repurposing candidates synergistic with immunotherapies

One major challenge in cancer immunotherapy is to increase tumor response to ICB treatment. Previous experimental and clinical studies have demonstrated that combinatorial therapeutic strategies could substantially increase the percentage of responder cases and contribute to significant survival benefits [38, 39]. A signature matching approach for drug prediction was adopted to discern more potential combination partners synergistic with immunotherapies (Fig. 6C). Since query signature was the basis of this approach, we first sought to collect potential immunotherapy-relevant genes for drug retrieval. Data from additional five ICB-treated screens were obtained. The experimental design used in these screens differed from ICB-naïve screens, which also introduced immunotherapeutic intervention into the experiments (Fig. S11A). Therefore, these screens could identify potential regulators mediating resistance (ICB enhancer genes) or sensitivity (ICB suppressor genes) to ICBs upon loss (Table S8). These screens studied four cancers (Fig. S11B) and common hits of enhancers and suppressors were also displayed, respectively (Fig. S11C). Since similar properties existed between sensitizers and enhancers as well as between resistors and suppressors, these genes with intersected (Fig. S11D, E). There were multiple common genes between the lists of sensitizers and enhancers (Fig. S11D). Subsequently, sensitizer genes, tumor immunity-related TSGs, and ICB enhancer genes were integrated into a meta-gene list, and genes in this list were termed positive regulators. Theoretically, potentiating the function of these genes might be related to an enhanced anti-tumor immune response. The list of negative regulators was obtained in the same way. Positive and negative regulators together constituted the query signature (Fig. 6c, Table S9).

The other necessary component of the signature matching-based approach was the drug signatures, also known as drug-induced profiles of expression changes. These drug signatures were downloaded from the Connectivity Map (CMap) datasets (CMap Build 2: 1288 compounds) [40]. To ensure easy clinical translation of our findings, we only selected drugs that have been approved or have already passed phase I and II clinical trials for subsequent analysis, leveraging the annotations from the Drug Repurposing Hub [41]. To match query signature with drug signatures, we applied three methods, including eXtreme Sum (XSum) [42], Kolmogorov-Smirnov (KS) [40], and the Reverse Gene Expression Score (RGES) [43]. Due to the varying measurement scales of the resultant scores from the three methods, an order statistics-based method developed by Stuart et al. was utilized to integrate these results and yielded a robust drug prediction result (Fig. 6C) [44]. The results of drug prediction are presented in Fig. 6D and Table S10. Notably, among the top 10 drugs, four candidates, including irinotecan, quercetin, trifluridine, and resveratrol, were previously reported by experimental or clinical studies, demonstrating the reliability of our prediction.

## Discussion

Compared to conventional anti-tumor strategies, immune-based therapeutics offer unique advantages and hold great promise for future cancer treatment. Despite the encouraging prospects, many problems have been encountered during the process of their clinical applications. Foremost among them is the de novo or acquired resistance to immunotherapeutic agents. Only a proportion of patients respond to immunotherapies and benefit from them. Even in such a small population of initial beneficiaries, some will ultimately develop immunotherapy resistance. To circumvent this complex issue, we considered the following three strategies: 1) developing more immuno-oncology targets; 2) constructing predictive signatures for patient selection; 3) designing combinatorial strategies to achieve synergistic antitumor activity. In this study, through integrative analyses of data from multiple screening experiments and large-scale clinical cohorts, we conducted preliminary explorations based on the above three strategies and obtained some promising findings, including a novel therapeutic target *MON2*, a predictive gene signature named CTIS, and some potential immunotherapeutic combinations.

*MON2* is a protein-coding gene that has been demonstrated to be involved in regulating endosome-to-Golgi trafficking [45]. To date, the relationship between *MON2* and the immune ecosystem remains largely unexplored. Our results suggested that the loss-of-function of *MON2* was associated with an enhanced response rate to ICBs, uncovering a new role of this gene associated with tumor immunity. Based on our findings, MON2 inhibition could be a potential therapeutic strategy with the ability to augment immune-induced tumor killing. Notably, the targets of most of the current immuno-oncology agents, such as PD-L1, CTLA-4, LAG-3 and TIM-3, express on the cell surface and can directly mediate tumor-immune interactions [46]. However, conceptually, *MON2* is not directly associated with immune-related functions, making it difficult to interpret its role as an immuno-oncology target. Similar to *MON2*, indoleamine-2,3-dioxygenase 1 (*IDO1*) is also a conceptually non-immune related gene, encoding heme enzyme for catalyzing the limiting step in tryptophan catabolism to N-formyl-kynurenine, which can exert immunosuppressive function via multiple mechanisms [47]. The inhibitors targeting *IDO1* have yielded some promising preclinical results, and many clinical trials on *IDO1* inhibitors are currently being conducted, which can give a rationale for taking *MON2* inhibition as a novel immunotherapeutic modality [47]. Currently, the *MON2* protein remains undruggable and cannot be targeted by conventional small-molecule inhibitors. Fortunately, the emergence of novel therapeutic approaches, such as antisense oligonucleotide (ASO) technologies and proteolysis-targeting chimeras (PROTACs), have enabled the pharmaceutical inhibition of *MON2* [48, 49].

The development of predictive biomarkers for patient selection for ICB treatment has been the focus of the present investigation. During the last few years, researchers have determined multiple biomarkers with predictive potential, including tumor mutation burden (TMB), MSI status, PD-1/PD-L1 gene expression, CD8^+^ T-cell abundance and et al. [29, 50]. Among these, TMB is the most studied biomarker; TMB-high has been considered as a universal indicator of high response rate of ICB treatment across cancers [51]. However, a very recent study found that TMB should only be considered an effective biomarker for ICB treatment in cancer types that exhibit positive correlation between CD8 cell abundance and neoantigens [52]. Accordingly, there exists an urgent need to develop more predictive biomarkers. Accumulating evidence indicates that transcriptome-based predictive signatures also perform well in predicting response to ICB treatment [20, 21, 53–56]. As an attempt, an expression-based signature was also developed in this study for predicting immunotherapeutic response. Although the overall performance of the CTIS signature was only slightly better than other established signatures, the relatively moderate correlation with other signatures suggested that CTIS was irreplaceable and might provide some additional prediction information.

Combined immunotherapy with other therapies, such as chemotherapy and targeted therapy, may enhance the tumor-killing effect compared to mono-administration. Since only some specific combinations exhibit synergistic effects, a key step in designing co-administration regimen is to identify the appropriate candidates for combination. Through in silico approaches, several efforts have been made to discern potential immunotherapeutic combinations [57, 58]. In the present study, we proposed an improved signature matching-based computational approach, which was the ensemble of multiple established approaches. This approach determined scores representing the possibility of synergizing cancer immunotherapy drugs. Remarkably, four of the top 10 drugs, including irinotecan, quercetin, trifluridine, and resveratrol, have been documented to have immunomodulatory functions. Irinotecan is a widely-used chemotherapeutic agent for treating multiple cancers. It has been reported that irinotecan could activate antitumor immunity via modulating the tumor microenvironment and enhance tumor immunogenicity by upregulating the expression of MHC class I genes, thereby synergizing with anti-PD-L1 therapies [59]. Quercetin is widely acknowledged to be an antioxidant flavonoid compound. In a recent study, the combination of quercetin with alantolactone was found to be capable of inducing synergistic immunogenic cell death (ICD), resulting in reactivation of antitumor immunity [60]. For trifluridine, as evidenced by a preclinical study, trifluridine/tipiracil (FTD/TPI) combined with anti-PD-1 monoclonal antibody exhibited greater antitumor activity against CMT-93 cells [61]. Among these four drugs, resveratrol has been most extensively studied for its immunomodulatory role. Briefly, resveratrol can enhance the antitumor immune response by inducing changes in the immune cell population [62, 63], regulating the secretion of inflammatory cytokines [64], and modulating the expression of immune checkpoint genes in tumor cells [65]. In general, the above evidence for these four drugs indirectly proved the rationality of our computational approach and the reliability of the prediction results.

## Conclusions

In conclusion, by leveraging extensive screening data as well as molecular and clinical data from TCGA and ICB-treated cohorts, this study determined a novel immunotherapeutic target, a predictive signature, and several candidate agents for immunotherapeutic combinations. Importantly, this study deepened our understanding of tumor immunity and provided the basis for future studies on cancer immunotherapies.

## Supplementary Information

**Additional file 1.** Supplemental Materials and Methods.

**Additional file 2: Table S1.** Summary of included screens. **Table S2.** The occurrence frequency of sensitizer and resistor genes in different cancer types. **Table S3.** Summary of 65 sensitizer genes and 40 resistor genes. **Table S4.** GO functional annotation of sensitizer and resistor genes. **Table S5.** The list of immune-related features. **Table S6.** Summary of studies associated with ICB treatment. **Table S7.** Summary of Tumor immunity-related OGs/TSGs. **Table S8.** Summary of ICB enhancer genes and ICB suppressor genes. **Table S9.** Summary of positive regulators and negative regulators. **Table S10.** Prediction of potential immunomodulatory agents.

**Additional file 3: Figure S1.** The distribution of 17 included screens which focused on investigating the regulators of immune cell-mediated killing among different screening types, library types, organisms, and algorithms. **Figure S2.** Results of Functional similarity analysis. **Figure S3.** Distribution of up- (red) and down- (blue) regulated genes (between tumor tissues and adjacent tissues) of sensitizers and resistors across different TCGA cancer types. **Figure S4.** The ratio between the inactivation event numbers of sensitizers and resistors. **Figure S5.** Functional characterization of sensitizers and resistors in each cancer type. **Figure S6.** Experimental validation of the function of MON2. **Figure S7.** The overall characterization of MON2. **Figure S8.** Relationship between MON2 and microsatellite instability (MSI). **Figure S9.** Comparison of the performance for predicting response to immunotherapy between CTIS and 14 published signatures in pretreatment datasets. **Figure S10.** Characterization of CTIS in TCGA Pan-Cancer cohort. **Figure S11.** Summary of ICB-treated CRISPR screens.

## Data Availability

All data generated or analyzed during this study are included in this published article and its supplementary data.

## Abbreviations

ICB: Immune checkpoint blockade
PD-1: Programmed cell death 1
PD-L1: Programmed cell death-ligand 1
CTLA-4: Cytotoxic T-lymphocyte antigen-4
FDA: Food and Drug Administration
CRISPR: Clustered regularly interspaced short palindromic repeats
CNV: Copy number variation
MSI: Microsatellite instability
GEO: Gene Expression Omnibus
ccRCC: Clear cell renal cell carcinoma
CR: Complete response
PR: Partial response
SD: Stable disease
PD: Progressive disease
DepMap: Dependency Map
CMap: Connectivity Map
sgRNAs: Single guide RNAs
FS: Functional similarity
GO: Gene Ontology
TSGs: Tumor suppressor genes
OGs: Oncogenes
XSum: eXtreme Sum
KS: Kolmogorov-Smirnov
RGES: Reverse Gene Expression Score
MD: Maximum deviation
RASs: Rank aggregation scores
PBMCs: Peripheral blood mononuclear cells
TIDE: Tracking of Indels by Decomposition
CRISPRko: CRISPR knockout
CTIS: CRISPR screening-based tumor-intrinsic immune score
CYT: Cytolytic activity
MSigDB: Molecular Signatures Database
OR: Odds ratio
SNV: Single nucleotide variant
ORR: Objective response rate
CMS: Consensus molecular subtypes
MSI-H: MSI-high
MSI-L: MSI-low
MSS: Microsatellite-stable
TMB: Tumor mutation burden
IDO1: Indoleamine-2,3-dioxygenase 1
ASO: Antisense oligonucleotide
PROTACs: Proteolysis-targeting chimeras; ICD: Immunogenic cell death

## References

[1] Callahan MK, Postow MA, Wolchok JD (2016). Targeting T cell co-receptors for Cancer therapy. Immunity..

[2] Hodi FS, O'Day SJ, McDermott DF, Weber RW, Sosman JA, Haanen JB, Gonzalez R, Robert C, Schadendorf D, Hassel JC (2010). Improved survival with ipilimumab in patients with metastatic melanoma. N Engl J Med.

[3] Brahmer J, Reckamp KL, Baas P, Crinò L, Eberhardt WE, Poddubskaya E, Antonia S, Pluzanski A, Vokes EE, Holgado E (2015). Nivolumab versus docetaxel in advanced squamous-cell non-small-cell lung Cancer. N Engl J Med.

[4] Garon EB, Rizvi NA, Hui R, Leighl N, Balmanoukian AS, Eder JP, Patnaik A, Aggarwal C, Gubens M, Horn L (2015). Pembrolizumab for the treatment of non-small-cell lung cancer. N Engl J Med.

[5] Akinleye A, Rasool Z (2019). Immune checkpoint inhibitors of PD-L1 as cancer therapeutics. J Hematol Oncol.

[6] Andrews LP, Marciscano AE, Drake CG, Vignali DA (2017). LAG3 (CD223) as a cancer immunotherapy target. Immunol Rev.

[7] Monney L, Sabatos CA, Gaglia JL, Ryu A, Waldner H, Chernova T, Manning S, Greenfield EA, Coyle AJ, Sobel RA (2002). Th1-specific cell surface protein Tim-3 regulates macrophage activation and severity of an autoimmune disease. Nature..

[8] Yu X, Harden K, Gonzalez LC, Francesco M, Chiang E, Irving B, Tom I, Ivelja S, Refino CJ, Clark H (2009). The surface protein TIGIT suppresses T cell activation by promoting the generation of mature immunoregulatory dendritic cells. Nat Immunol.

[9] Sharma P, Hu-Lieskovan S, Wargo JA, Ribas A (2017). Primary, adaptive, and acquired resistance to Cancer immunotherapy. Cell..

[10] Patel SJ, Sanjana NE, Kishton RJ, Eidizadeh A, Vodnala SK, Cam M, Gartner JJ, Jia L, Steinberg SM, Yamamoto TN (2017). Identification of essential genes for cancer immunotherapy. Nature..

[11] Manguso RT, Pope HW, Zimmer MD, Brown FD, Yates KB, Miller BC, Collins NB, Bi K, LaFleur MW, Juneja VR (2017). In vivo CRISPR screening identifies Ptpn2 as a cancer immunotherapy target. Nature..

[12] Lawson KA, Sousa CM, Zhang X, Kim E, Akthar R, Caumanns JJ, Yao Y, Mikolajewicz N, Ross C, Brown KR (2020). Functional genomic landscape of cancer-intrinsic evasion of killing by T cells. Nature..

[13] Dubrot J, Lane-Reticker SK, Kessler EA, Ayer A, Mishra G, Wolfe CH, Zimmer MD, Du PP, Mahapatra A, Ockerman KM (2021). In vivo screens using a selective CRISPR antigen removal lentiviral vector system reveal immune dependencies in renal cell carcinoma. Immunity..

[14] Miles LA, Garippa RJ, Poirier JT (2016). Design, execution, and analysis of pooled in vitro CRISPR/Cas9 screens. FEBS J.

[15] Liu D, Zhao X, Tang A, Xu X, Liu S, Zha L, Ma W, Zheng J, Shi M (2020). CRISPR screen in mechanism and target discovery for cancer immunotherapy. Biochim Biophys Acta Rev Cancer.

[16] Wang B, Wang M, Zhang W, Xiao T, Chen CH, Wu A, Wu F, Traugh N, Wang X, Li Z (2019). Integrative analysis of pooled CRISPR genetic screens using MAGeCKFlute. Nat Protoc.

[17] Adzhubei IA, Schmidt S, Peshkin L, Ramensky VE, Gerasimova A, Bork P, Kondrashov AS, Sunyaev SR (2010). A method and server for predicting damaging missense mutations. Nat Methods.

[18] Flanagan SE, Patch AM, Ellard S (2010). Using SIFT and PolyPhen to predict loss-of-function and gain-of-function mutations. Genet Test Mol Biomarkers.

[19] Yoshihara K, Shahmoradgoli M, Martínez E, Vegesna R, Kim H, Torres-Garcia W, Treviño V, Shen H, Laird PW, Levine DA (2013). Inferring tumour purity and stromal and immune cell admixture from expression data. Nat Commun.

[20] Rooney MS, Shukla SA, Wu CJ, Getz G, Hacohen N (2015). Molecular and genetic properties of tumors associated with local immune cytolytic activity. Cell..

[21] Lauss M, Donia M, Harbst K, Andersen R, Mitra S, Rosengren F, Salim M, Vallon-Christersson J, Törngren T, Kvist A (2017). Mutational and putative neoantigen load predict clinical benefit of adoptive T cell therapy in melanoma. Nat Commun.

[22] Weinstein JN, Collisson EA, Mills GB, Shaw KR, Ozenberger BA, Ellrott K, Shmulevich I, Sander C, Stuart JM (2013). The Cancer genome atlas Pan-Cancer analysis project. Nat Genet.

[23] Li Y, Burgman B, McGrail DJ, Sun M, Qi D, Shukla SA, Wu E, Capasso A, Lin SY, Wu CJ (2020). Integrated genomic characterization of the human Immunome in Cancer. Cancer Res.

[24] Liberzon A, Subramanian A, Pinchback R, Thorvaldsdóttir H, Tamayo P, Mesirov JP (2011). Molecular signatures database (MSigDB) 3.0. Bioinformatics..

[25] Thorsson V, Gibbs DL, Brown SD, Wolf D, Bortone DS, Ou Yang TH, Porta-Pardo E, Gao GF, Plaisier CL, Eddy JA (2018). The immune landscape of Cancer. Immunity..

[26] Bhattacharya S, Dunn P, Thomas CG, Smith B, Schaefer H, Chen J, Hu Z, Zalocusky KA, Shankar RD, Shen-Orr SS (2018). ImmPort, toward repurposing of open access immunological assay data for translational and clinical research. Scientific Data.

[27] Tsherniak A, Vazquez F, Montgomery PG, Weir BA, Kryukov G, Cowley GS, Gill S, Harrington WF, Pantel S, Krill-Burger JM (2017). Defining a Cancer dependency map. Cell..

[28] Brinkman EK, Chen T, Amendola M, van Steensel B (2014). Easy quantitative assessment of genome editing by sequence trace decomposition. Nucleic Acids Res.

[29] Lee JS, Ruppin E (2019). Multiomics prediction of response rates to therapies to inhibit programmed cell death 1 and programmed cell death 1 ligand 1. JAMA Oncol.

[30] Xiao Y, Freeman GJ (2015). The microsatellite instable subset of colorectal cancer is a particularly good candidate for checkpoint blockade immunotherapy. Cancer Discov.

[31] Cortes-Ciriano I, Lee S, Park WY, Kim TM, Park PJ (2017). A molecular portrait of microsatellite instability across multiple cancers. Nat Commun.

[32] Chang L, Chang M, Chang HM, Chang F (2018). Microsatellite instability: a predictive biomarker for Cancer immunotherapy. Appl Immunohistochem Mol Morphol.

[33] Guinney J, Dienstmann R, Wang X, de Reyniès A, Schlicker A, Soneson C, Marisa L, Roepman P, Nyamundanda G, Angelino P (2015). The consensus molecular subtypes of colorectal cancer. Nat Med.

[34] Liu D, Schilling B, Liu D, Sucker A, Livingstone E, Jerby-Arnon L, Zimmer L, Gutzmer R, Satzger I, Loquai C (2019). Integrative molecular and clinical modeling of clinical outcomes to PD1 blockade in patients with metastatic melanoma. Nat Med.

[35] Wellenstein MD, de Visser KE (2018). Cancer-cell-intrinsic mechanisms shaping the tumor immune landscape. Immunity..

[36] Casey SC, Baylot V, Felsher DW (2018). The MYC oncogene is a global regulator of the immune response. Blood..

[37] Chakravarty D, Gao J, Phillips SM, Kundra R, Zhang H, Wang J, et al. OncoKB: a precision oncology Knowledge Base. JCO Precis Oncol. https://ascopubs.org/doi/10.1200/PO.17.00011. 10.1200/PO.17.00011 PMC5586540 28890946

[38] Yu WD, Sun G, Li J, Xu J, Wang X (2019). Mechanisms and therapeutic potentials of cancer immunotherapy in combination with radiotherapy and/or chemotherapy. Cancer Lett.

[39] Finn RS, Qin S, Ikeda M, Galle PR, Ducreux M, Kim TY, Kudo M, Breder V, Merle P, Kaseb AO (2020). Atezolizumab plus Bevacizumab in Unresectable Hepatocellular Carcinoma. N Engl J Med.

[40] Lamb J, Crawford ED, Peck D, Modell JW, Blat IC, Wrobel MJ, Lerner J, Brunet JP, Subramanian A, Ross KN (2006). The connectivity map: using gene-expression signatures to connect small molecules, genes, and disease. Science..

[41] Corsello SM, Bittker JA, Liu Z, Gould J, McCarren P, Hirschman JE, Johnston SE, Vrcic A, Wong B, Khan M (2017). The drug repurposing hub: a next-generation drug library and information resource. Nat Med.

[42] Cheng J, Yang L, Kumar V, Agarwal P (2014). Systematic evaluation of connectivity map for disease indications. Genome Med.

[43] Chen B, Ma L, Paik H, Sirota M, Wei W, Chua MS, So S, Butte AJ (2017). Reversal of cancer gene expression correlates with drug efficacy and reveals therapeutic targets. Nat Commun.

[44] Stuart JM, Segal E, Koller D, Kim SK (2003). A gene-coexpression network for global discovery of conserved genetic modules. Science..

[45] Mahajan D, Boh BK, Zhou Y, Chen L, Cornvik TC, Hong W, Lu L (2013). Mammalian Mon2/Ysl2 regulates endosome-to-Golgi trafficking but possesses no guanine nucleotide exchange activity toward Arl1 GTPase. Sci Rep.

[46] Qin S, Xu L, Yi M, Yu S, Wu K, Luo S (2019). Novel immune checkpoint targets: moving beyond PD-1 and CTLA-4. Mol Cancer.

[47] Le Naour J, Galluzzi L, Zitvogel L, Kroemer G, Vacchelli E (2020). Trial watch: IDO inhibitors in cancer therapy. Oncoimmunology..

[48] Kole R, Krainer AR, Altman S (2012). RNA therapeutics: beyond RNA interference and antisense oligonucleotides. Nat Rev Drug Discov.

[49] Burslem GM, Crews CM (2020). Proteolysis-targeting chimeras as therapeutics and tools for biological discovery. Cell..

[50] Pender A, Titmuss E, Pleasance ED, Fan KY, Pearson H, Brown SD, Grisdale CJ, Topham JT, Shen Y, Bonakdar M (2021). Genome and transcriptome biomarkers of response to immune checkpoint inhibitors in advanced solid tumors. Clin Cancer Res.

[51] Marabelle A, Fakih M, Lopez J, Shah M, Shapira-Frommer R, Nakagawa K, Chung HC, Kindler HL, Lopez-Martin JA, Miller WH (2020). Association of tumour mutational burden with outcomes in patients with advanced solid tumours treated with pembrolizumab: prospective biomarker analysis of the multicohort, open-label, phase 2 KEYNOTE-158 study. Lancet Oncol.

[52] McGrail DJ, Pilié PG, Rashid NU, Voorwerk L, Slagter M, Kok M, et al. High tumor mutation burden fails to predict immune checkpoint blockade response across all cancer types. Ann Oncol. 2021;32:661–72. 10.1016/j.annonc.2021.02.006 PMC8053682 33736924

[53] Jiang P, Gu S, Pan D, Fu J, Sahu A, Hu X, Li Z, Traugh N, Bu X, Li B (2018). Signatures of T cell dysfunction and exclusion predict cancer immunotherapy response. Nat Med.

[54] Messina JL, Fenstermacher DA, Eschrich S, Qu X, Berglund AE, Lloyd MC, Schell MJ, Sondak VK, Weber JS, Mulé JJ (2012). 12-chemokine gene signature identifies lymph node-like structures in melanoma: potential for patient selection for immunotherapy?. Sci Rep.

[55] Ayers M, Lunceford J, Nebozhyn M, Murphy E, Loboda A, Kaufman DR, Albright A, Cheng JD, Kang SP, Shankaran V (2017). IFN-γ-related mRNA profile predicts clinical response to PD-1 blockade. J Clin Invest.

[56] Hugo W, Zaretsky JM, Sun L, Song C, Moreno BH, Hu-Lieskovan S, Berent-Maoz B, Pang J, Chmielowski B, Cherry G (2016). Genomic and transcriptomic features of response to anti-PD-1 therapy in metastatic melanoma. Cell..

[57] Gu SS, Zhang W, Wang X, Jiang P, Traugh N, Li Z, et al. Therapeutically increasing MHC-I expression potentiates immune checkpoint blockade. Cancer Discov. 2021;11:1524–41. 10.1158/2159-8290.CD-20-0812 PMC8543117 33589424

[58] Lesterhuis WJ, Rinaldi C, Jones A, Rozali EN, Dick IM, Khong A, Boon L, Robinson BW, Nowak AK, Bosco A (2015). Network analysis of immunotherapy-induced regressing tumours identifies novel synergistic drug combinations. Sci Rep.

[59] Iwai T, Sugimoto M, Wakita D, Yorozu K, Kurasawa M, Yamamoto K (2018). Topoisomerase I inhibitor, irinotecan, depletes regulatory T cells and up-regulates MHC class I and PD-L1 expression, resulting in a supra-additive antitumor effect when combined with anti-PD-L1 antibodies. Oncotarget..

[60] Zhang J, Shen L, Li X, Song W, Liu Y, Huang L (2019). Nanoformulated Codelivery of quercetin and Alantolactone promotes an antitumor response through synergistic immunogenic cell death for microsatellite-stable colorectal Cancer. ACS Nano.

[61] Suzuki N, Tsukihara H, Nakagawa F, Kobunai T, Takechi T (2017). Synergistic anticancer activity of a novel oral chemotherapeutic agent containing trifluridine and tipiracil in combination with anti-PD-1 blockade in microsatellite stable-type murine colorectal cancer cells. Am J Cancer Res.

[62] Yang Y, Paik JH, Cho D, Cho JA, Kim CW (2008). Resveratrol induces the suppression of tumor-derived CD4+CD25+ regulatory T cells. Int Immunopharmacol.

[63] Chen L, Yang S, Liao W, Xiong Y (2015). Modification of antitumor immunity and tumor microenvironment by resveratrol in mouse renal tumor model. Cell Biochem Biophys.

[64] Bergman M, Levin GS, Bessler H, Djaldetti M, Salman H (2013). Resveratrol affects the cross talk between immune and colon cancer cells. Biomed Pharmacother.

[65] Lucas J, Hsieh TC, Halicka HD, Darzynkiewicz Z, Wu JM (2018). Upregulation of PD-L1 expression by resveratrol and piceatannol in breast and colorectal cancer cells occurs via HDAC3/p300-mediated NF-κB signaling. Int J Oncol.

