# Supplementary material for: Integrative analysis of CRISPR screening data uncovers new opportunities for optimizing cancer immunotherapy
Source: Mol Cancer. 2022 Jan 2;21:2. doi: 10.1186/s12943-021-01462-z (PMC8722047; doi:10.1186/s12943-021-01462-z)
Supplement: Supplementary file 1 — Additional file 1. Supplemental Materials and Methods. [file 12943_2021_1462_MOESM1_ESM.docx]

**Supplemental Materials and Methods**

**Treatment-****naïve clinical data**

Multi-omics data, including normalized and batch corrected gene expression data and gene mutation data, generated by the PanCancer Atlas consortium, were downloaded from the publication page ([gdc.cancer.gov/about-data/publications/pancanatlas](https://gdc.cancer.gov/about-data/publications/pancanatlas)) [1]. Data of gene-level copy number variation (CNV) estimated using the GISTIC2 was obtained from UCSC Xena ([xenabrowser.net](https://xenabrowser.net)). Survival data corresponding to the samples with molecular data were achieved from TCGA Pan-Cancer Clinical Data Resource (TCGA-CDR) [2]. Immune cell infiltration data and annotation information of immune subtypes were assessed from the supplementary file of the reference [3]. Microsatellite instability (MSI) status and the number of MSI events of TCGA samples were also obtained from the supplementary file of previous publication [4].

**ICB-treated clinical data**

Expression and mutation (only partially available) data as well as corresponding clinical data of eight immune-checkpoint blockade (ICB)-treated datasets, which included advanced melanoma patients treated with anti-PD-L1 therapy [5], melanoma patients treated with anti-CTLA-4 therapy [6], metastatic melanoma patients treated with anti-CTLA-4 therapy [7], clear cell renal cell carcinoma (ccRCC) patients treated with various types of immunotherapy [8], metastatic melanoma patients treated with anti-PD-L1 therapy [9, 10], urothelial cancer patients treated with anti-PD-L1 therapy [11, 12], were obtained from corresponding studies. Batch effects between different datasets were removed using the *ComBat* function from *sva* R package [13]. Here, patients who achieved a complete response (CR) or partial response (PR) were categorized as responders, and non-responders were defined as those patients who displayed stable (SD) or progressive disease (PD).

**Cancer cell line data**

Genome-wide gene dependency data (CERES scores from CRISPR knockout screens) across 739 cancer cell lines were achieved from the Cancer Dependency Map (DepMap) portal (20Q1, released February 2020) [14]. Mean CERES scores for each gene were calculated. Lower CERES scores indicated that corresponding genes are more likely to be essential in cell growth and survival. Merged compound-induced expression profiles of three cancer cell lines (MCF7, PC3, and HL60) were downloaded from the Connectivity Map (CMap) datasets (CMap Build 2: 1,288 compounds) [15].

**Functional Similarity Analysis**

We calculated the functional similarity (FS) scores between gene pairs through utilizing the semantic similarities in molecular function and cellular component aspect of Gene Ontology (GO) terms. This approach takes both function and location of genes into account which can achieve a comprehensive characterization of gene function [16]. The FS score for a certain gene pair is given as:

FS score = $\sqrt{SimMF*SimCC}$

Semantic similarities in MF (SimMF) and CC (SimCC) could be measured based on the GO topological structure using *GOSemSim* package [17].

**Predictive signature construction**

The predictive signature was constructed based on 105 sensitizer/resistor genes. Considering that the properties of tumors could be greatly different between baseline samples and on-treatment samples, we only utilized baseline samples for signature construction and validation. A preliminary filtering based on S-/R-related feature numbers was first carried out and only genes with more than 100 related features were kept for the subsequent analysis. Random survival forest (RSF) algorithm was then performed using the *randomForestSRC* R package to further narrow down the gene list [18]. In RSF analysis, 500 trees were grown using a log-rank, score-splitting algorithm; variables were ranked by minimal depth (MD), of which smaller value indicated greater predictiveness. The RSF analysis was independently repeated 1000 times and the concordance index (C-index) in out-of-bag (OOB) samples of each iteration was recorded. Gene combination with largest C-index were considered as the optimal candidates. This strategy was also adopted by our previous publication [19]. We then defined the CRISPR screening-based tumor-intrinsic immune score (CTIS) as follows:

CTIS = $\frac{1}{Number (S)}\sum Exp(Sensitizers)-\frac{1}{Number (R)}\sum Exp(Resistors)$

Conceptually, a higher CTIS indicated a stronger anti-tumor immune response.

Many previously published immune signatures were also included for comparison. To ensure a comprehensive coverage of classical immune signatures, several studies which provided the summary of immune signatures were referenced [20-22]. As a result, a total of 14 published signatures were included in this study, including CD8 Sig [21], Chemokine Sig [23], CYT Sig [24], DDR Sig [25, 26], IFN Sig1 [27], IFN Sig2 [24], IFNG Sig [26], Inflamed Sig [26], IPRES Sig [9], MHC Sig [28], PD-L1 Sig [21], Tcell Sig1 [29], Tcell Sig2 [24] and Stress Sig [30].

**Signature matching**

Three different methods, including eXtreme Sum (XSum) [31], Kolmogorov-Smirnov (KS) [15], and the Reverse Gene Expression Score (RGES) [32] were adopted to perform signature matching analysis.

The XSum method handles the positive regulators and negative regulators separately. In brief, the sums of the change values in drug signatures relative to positive regulators (Sum_pos_) and negative regulators (Sum_neg_) are first calculated. Then, XSum is defined as following (the topN of XSum was set at 500): XSum = Sum_pos_ − Sum_neg_. The KS method has been the most widely used method for signature matching [15]. Similar to XSum, the KS method also needs to separate query signatures into two gene sets, namely positive regulators and negative regulators in this study. Briefly, using complete drug profiles as reference, maximum deviation (MD)-based enrichment scores of positive regulators (ES_pos_) and negative regulators (ES_neg_) should first be computed. If ES_pos_ and ES_neg_ have the same algebraic sign then KSscore = 0, otherwise, KSscore = ES_pos_ − ES_neg_. The RGES method is a modification of the original KS method, which has been demonstrated to have a better performance in drug prediction than the KS method by a recent publication [32]. In contrast to KS method, RGES focused on the reversal relation between the disease and agents, and RGES is defined as ES_pos_ − ES_neg_ regardless of the sign of ES_pos_ and ES_neg_. Additionally, there also existed some differences in other details between KS and RGES [32].

To obtain a consistent result across these methods, an order statistics-based rank aggregation approach proposed by Stuart *et al.* was used in this study [33]. The output scores of this approach were probabilities. The rank aggregation scores (RASs) were then defined as follows:

RAS = −log_10_(Probability score).

A final ranking list of candidate drugs could then be determined based on the resultant RASs, and a higher RAS indicated a more concordant high ranking.

**Isolation and generation of Mart-1 specific T cells**

Peripheral blood mononuclear cells (PBMCs) were isolated from healthy donor buffy coats (Heidelberg, Germany) through density gradient centrifugation using FICOLL PAQUE PLUS (GE health). CD3 T cells were purified and activated using aCD3/aCD28 Dynabeads (Invitrogen) following the manufacturer’s instructions. Activated T cells were then transduced with a TCR specific for Mart-1 using spinfection. Mart-1 – specific T cells were cultured for 7 days before the coculture experiment. T cells were cultured in RPMI containing 10% FBS (GIBCO), 100 units/ml of penicillin, 100 ug/ml of streptomycin, 2mM Glutamax (Gibco), 100 units/ml IL-2, and 10ng/ml IL-15 (PeproTech).

**Tumor cell - T cell coculture assays**

Tumor cells were seeded in 96-well culture plates, 1 x 10^4^/well. After 24 hours, Mart-1-specific T cells were added to tumor cells at T cell: tumor cell ratios -1:1 for MDA-MB-231 and 4:1 for MCF7. After 24 hours (for MDA-MB-231) or 16 hours (for MCF7), T cells were washed away. Tumor cell viability was determined using CellTiter Blue assay (Promega) following the manufacturer’s instructions. After incubation at 37℃ for 1-2 hours. The fluorescence of the solution was measured on an Infinite 200 Pro spectrophotometer (Tecan) at 535nm.

**Generation of knockout cell lines**

Three knockout cell lines were obtained by CRISPR/Cas9 gene knockout system with three independent sgRNAs against *MON2*. To determine the CRISPR knockout efficiency, Tracking of Indels by Decomposition (TIDE) analysis was performed following the developer's instruction. Briefly, PCR products amplified from genomic DNA of wide type and sgRNA transduced cells were sequenced. The sequencing data served as input for the TIDE analysis (<https://tide-calculator.nki.nl/>).

**References**

1. Weinstein JN, Collisson EA, Mills GB, Shaw KR, Ozenberger BA, Ellrott K, Shmulevich I, Sander C, Stuart JM. The Cancer Genome Atlas Pan-Cancer analysis project. Nat Genet. 2013;45:1113-20.

2. Liu J, Lichtenberg T, Hoadley KA, Poisson LM, Lazar AJ, Cherniack AD, Kovatich AJ, Benz CC, Levine DA, Lee AV, et al. An Integrated TCGA Pan-Cancer Clinical Data Resource to Drive High-Quality Survival Outcome Analytics. Cell. 2018;173:400-16.e11.

3. Thorsson V, Gibbs DL, Brown SD, Wolf D, Bortone DS, Ou Yang TH, Porta-Pardo E, Gao GF, Plaisier CL, Eddy JA, et al. The Immune Landscape of Cancer. Immunity. 2018;48:812-30.e14.

4. Cortes-Ciriano I, Lee S, Park WY, Kim TM, Park PJ. A molecular portrait of microsatellite instability across multiple cancers. Nature communications. 2017;8:15180.

5. Riaz N, Havel JJ, Makarov V, Desrichard A, Urba WJ, Sims JS, Hodi FS, Martín-Algarra S, Mandal R, Sharfman WH, et al. Tumor and Microenvironment Evolution during Immunotherapy with Nivolumab. Cell. 2017;171:934-49.e16.

6. Nathanson T, Ahuja A, Rubinsteyn A, Aksoy BA, Hellmann MD, Miao D, Van Allen E, Merghoub T, Wolchok JD, Snyder A, et al. Somatic Mutations and Neoepitope Homology in Melanomas Treated with CTLA-4 Blockade. Cancer immunology research. 2017;5:84-91.

7. Van Allen EM, Miao D, Schilling B, Shukla SA, Blank C, Zimmer L, Sucker A, Hillen U, Foppen MHG, Goldinger SM, et al. Genomic correlates of response to CTLA-4 blockade in metastatic melanoma. Science. 2015;350:207-11.

8. Miao D, Margolis CA, Gao W, Voss MH, Li W, Martini DJ, Norton C, Bossé D, Wankowicz SM, Cullen D, et al. Genomic correlates of response to immune checkpoint therapies in clear cell renal cell carcinoma. Science. 2018;359:801-6.

9. Hugo W, Zaretsky JM, Sun L, Song C, Moreno BH, Hu-Lieskovan S, Berent-Maoz B, Pang J, Chmielowski B, Cherry G, et al. Genomic and Transcriptomic Features of Response to Anti-PD-1 Therapy in Metastatic Melanoma. Cell. 2016;165:35-44.

10. Liu D, Schilling B, Liu D, Sucker A, Livingstone E, Jerby-Arnon L, Zimmer L, Gutzmer R, Satzger I, Loquai C, et al. Integrative molecular and clinical modeling of clinical outcomes to PD1 blockade in patients with metastatic melanoma. Nat Med. 2019;25:1916-27.

11. Mariathasan S, Turley SJ, Nickles D, Castiglioni A, Yuen K, Wang Y, Kadel EE, III, Koeppen H, Astarita JL, Cubas R, et al. TGFβ attenuates tumour response to PD-L1 blockade by contributing to exclusion of T cells. Nature. 2018;554:544-8.

12. Snyder A, Nathanson T, Funt SA, Ahuja A, Buros Novik J, Hellmann MD, Chang E, Aksoy BA, Al-Ahmadie H, Yusko E, et al. Contribution of systemic and somatic factors to clinical response and resistance to PD-L1 blockade in urothelial cancer: An exploratory multi-omic analysis. PLoS Med. 2017;14:e1002309.

13. Leek JT, Johnson WE, Parker HS, Jaffe AE, Storey JD. The sva package for removing batch effects and other unwanted variation in high-throughput experiments. Bioinformatics. 2012;28:882-3.

14. Tsherniak A, Vazquez F, Montgomery PG, Weir BA, Kryukov G, Cowley GS, Gill S, Harrington WF, Pantel S, Krill-Burger JM, et al. Defining a Cancer Dependency Map. Cell. 2017;170:564-76.e16.

15. Lamb J, Crawford ED, Peck D, Modell JW, Blat IC, Wrobel MJ, Lerner J, Brunet JP, Subramanian A, Ross KN, et al. The Connectivity Map: using gene-expression signatures to connect small molecules, genes, and disease. Science. 2006;313:1929-35.

16. Han Y, Yu G, Sarioglu H, Caballero-Martinez A, Schlott F, Ueffing M, Haase H, Peschel C, Krackhardt AM. Proteomic investigation of the interactome of FMNL1 in hematopoietic cells unveils a role in calcium-dependent membrane plasticity. J Proteomics. 2013;78:72-82.

17. Yu G, Li F, Qin Y, Bo X, Wu Y, Wang S. GOSemSim: an R package for measuring semantic similarity among GO terms and gene products. Bioinformatics. 2010;26:976-8.

18. Chen X, Ishwaran H. Random forests for genomic data analysis. Genomics. 2012;99:323-9.

19. Yang C, Huang X, Li Y, Chen J, Lv Y, Dai S. Prognosis and personalized treatment prediction in TP53-mutant hepatocellular carcinoma: an in silico strategy towards precision oncology. Brief Bioinform. 2021;22.

20. Xiong D, Wang Y, You M. A gene expression signature of TREM2(hi) macrophages and γδ T cells predicts immunotherapy response. Nat Commun. 2020;11:5084.

21. Jiang P, Gu S, Pan D, Fu J, Sahu A, Hu X, Li Z, Traugh N, Bu X, Li B, et al. Signatures of T cell dysfunction and exclusion predict cancer immunotherapy response. Nat Med. 2018;24:1550-8.

22. Hu FF, Liu CJ, Liu LL, Zhang Q, Guo AY. Expression profile of immune checkpoint genes and their roles in predicting immunotherapy response. Brief Bioinform. 2021;22.

23. Messina JL, Fenstermacher DA, Eschrich S, Qu X, Berglund AE, Lloyd MC, Schell MJ, Sondak VK, Weber JS, Mulé JJ. 12-Chemokine gene signature identifies lymph node-like structures in melanoma: potential for patient selection for immunotherapy? Sci Rep. 2012;2:765.

24. Rooney MS, Shukla SA, Wu CJ, Getz G, Hacohen N. Molecular and genetic properties of tumors associated with local immune cytolytic activity. Cell. 2015;160:48-61.

25. Conway JR, Kofman E, Mo SS, Elmarakeby H, Van Allen E. Genomics of response to immune checkpoint therapies for cancer: implications for precision medicine. Genome Med. 2018;10:93.

26. Ayers M, Lunceford J, Nebozhyn M, Murphy E, Loboda A, Kaufman DR, Albright A, Cheng JD, Kang SP, Shankaran V, et al. IFN-γ-related mRNA profile predicts clinical response to PD-1 blockade. J Clin Invest. 2017;127:2930-40.

27. Danilova L, Ho WJ, Zhu Q, Vithayathil T, De Jesus-Acosta A, Azad NS, Laheru DA, Fertig EJ, Anders R, Jaffee EM, et al. Programmed Cell Death Ligand-1 (PD-L1) and CD8 Expression Profiling Identify an Immunologic Subtype of Pancreatic Ductal Adenocarcinomas with Favorable Survival. Cancer Immunol Res. 2019;7:886-95.

28. Lauss M, Donia M, Harbst K, Andersen R, Mitra S, Rosengren F, Salim M, Vallon-Christersson J, Törngren T, Kvist A, et al. Mutational and putative neoantigen load predict clinical benefit of adoptive T cell therapy in melanoma. Nature communications. 2017;8:1738.

29. Azizi E, Carr AJ, Plitas G, Cornish AE, Konopacki C, Prabhakaran S, Nainys J, Wu K, Kiseliovas V, Setty M, et al. Single-Cell Map of Diverse Immune Phenotypes in the Breast Tumor Microenvironment. Cell. 2018;174:1293-308.e36.

30. Li H, van der Leun AM, Yofe I, Lubling Y, Gelbard-Solodkin D, van Akkooi ACJ, van den Braber M, Rozeman EA, Haanen J, Blank CU, et al. Dysfunctional CD8 T Cells Form a Proliferative, Dynamically Regulated Compartment within Human Melanoma. Cell. 2019;176:775-89.e18.

31. Cheng J, Yang L, Kumar V, Agarwal P. Systematic evaluation of connectivity map for disease indications. Genome Med. 2014;6:540.

32. Chen B, Ma L, Paik H, Sirota M, Wei W, Chua MS, So S, Butte AJ. Reversal of cancer gene expression correlates with drug efficacy and reveals therapeutic targets. Nature communications. 2017;8:16022.

33. Stuart JM, Segal E, Koller D, Kim SK. A gene-coexpression network for global discovery of conserved genetic modules. Science. 2003;302:249-55.
