# Supplementary material for: Integrative analysis of CRISPR screening data uncovers new opportunities for optimizing cancer immunotherapy
Source: Mol Cancer. 2022 Jan 2;21:2. doi: 10.1186/s12943-021-01462-z (PMC8722047; doi:10.1186/s12943-021-01462-z)
Supplement: Supplementary file 3 — Additional file 3: Figure S1. The distribution of 17 included screens which focused on investigating the regulators of immune cell-mediated killing among different screening types, library types, organisms, and algorithms. Figure S2. Results of Functional similarity analysis. Figure S3. Distribution of up- (red) and down- (blue) regulated genes (between tumor tissues and adjacent tissues) of sensitizers and resistors across different TCGA cancer types. Figure S4. The ratio between the inactivation event numbers of sensitizers and resistors. Figure S5. Functional characterization of sensitizers and resistors in each cancer type. Figure S6. Experimental validation of the function of MON2. Figure S7. The overall characterization of MON2. Figure S8. Relationship between MON2 and microsatellite instability (MSI). Figure S9. Comparison of the performance for predicting response to immunotherapy between CTIS and 14 published signatures in pretreatment datasets. Figure S10. Characterization of CTIS in TCGA Pan-Cancer cohort. Figure S11. Summary of ICB-treated CRISPR screens. [file 12943_2021_1462_MOESM3_ESM.pdf]

Supplementary Figures

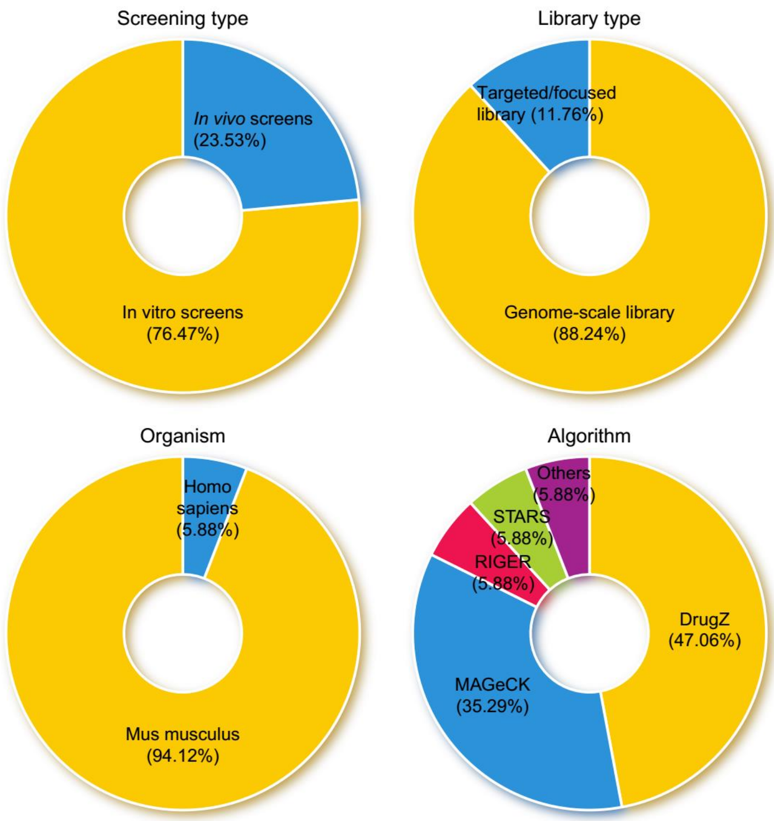

Figure S1. The distribution of 17 included screens which focused on investigating the regulators of immune cell-meditated killing among different screening types, library types, organisms, and algorithms.

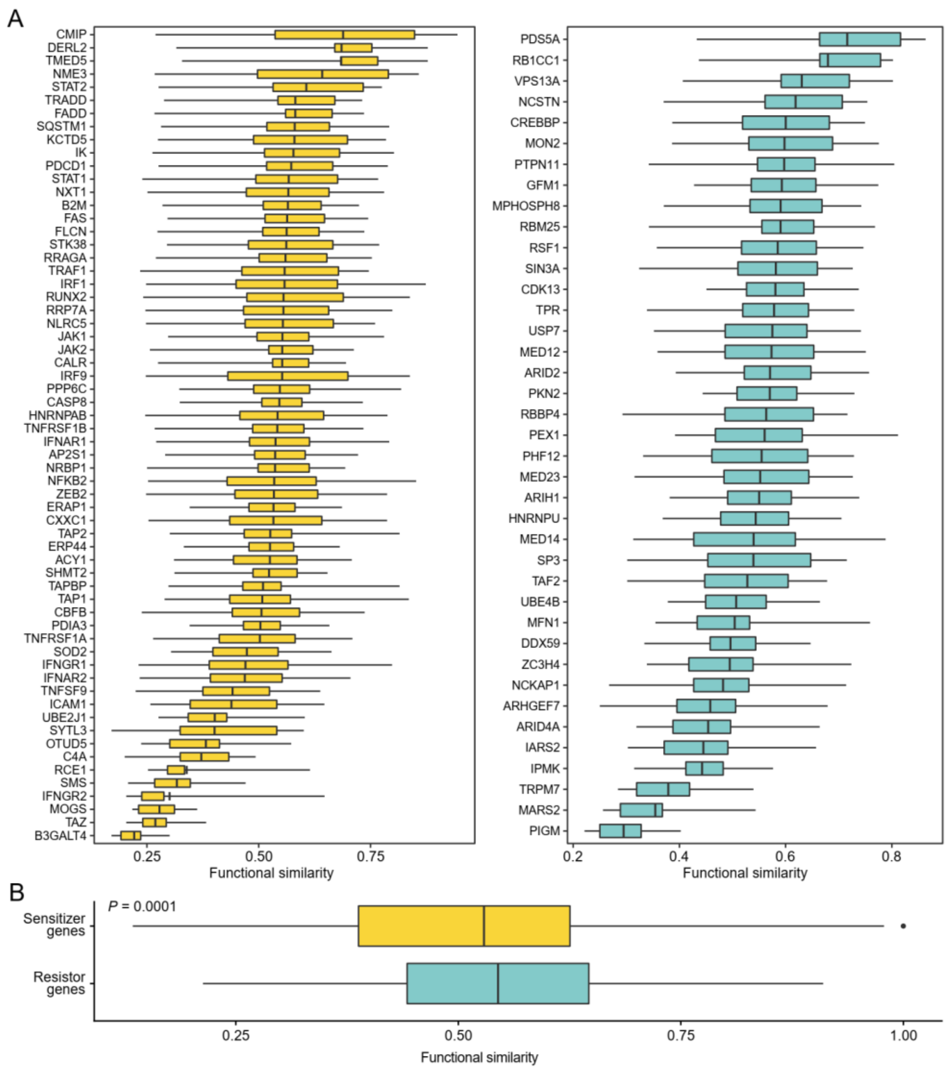

Figure S2. Results of Functional similarity analysis. (A) Summary of functional similarities of 65 sensitizer genes (left) and 40 resistor genes (right). The boxplots were used to show the distributions of functional similarities. (B) Comparison of functional similarities between sensitizer and resistor genes. Statistical significance of difference was determined using Wilcoxon rank-sum test.

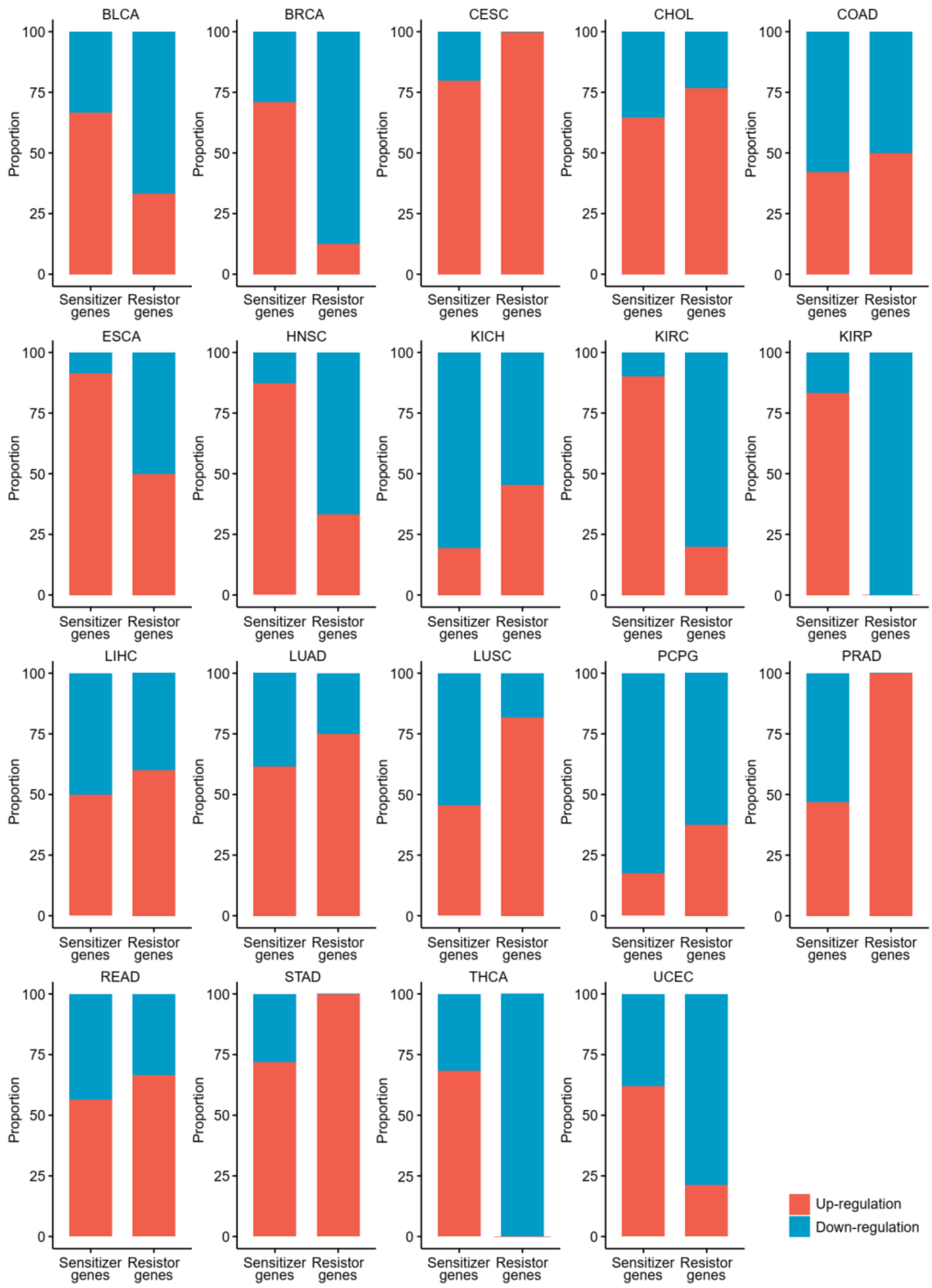

Figure S3. Distribution of up- (red) and down- (blue) regulated genes (between tumor tissues and adjacent tissues) of sensitizers and resistors across different TCGA cancer types. Only genes with adjusted $P < 0.05$ and abs(log Fold change) > 0.5 were included into statistics.

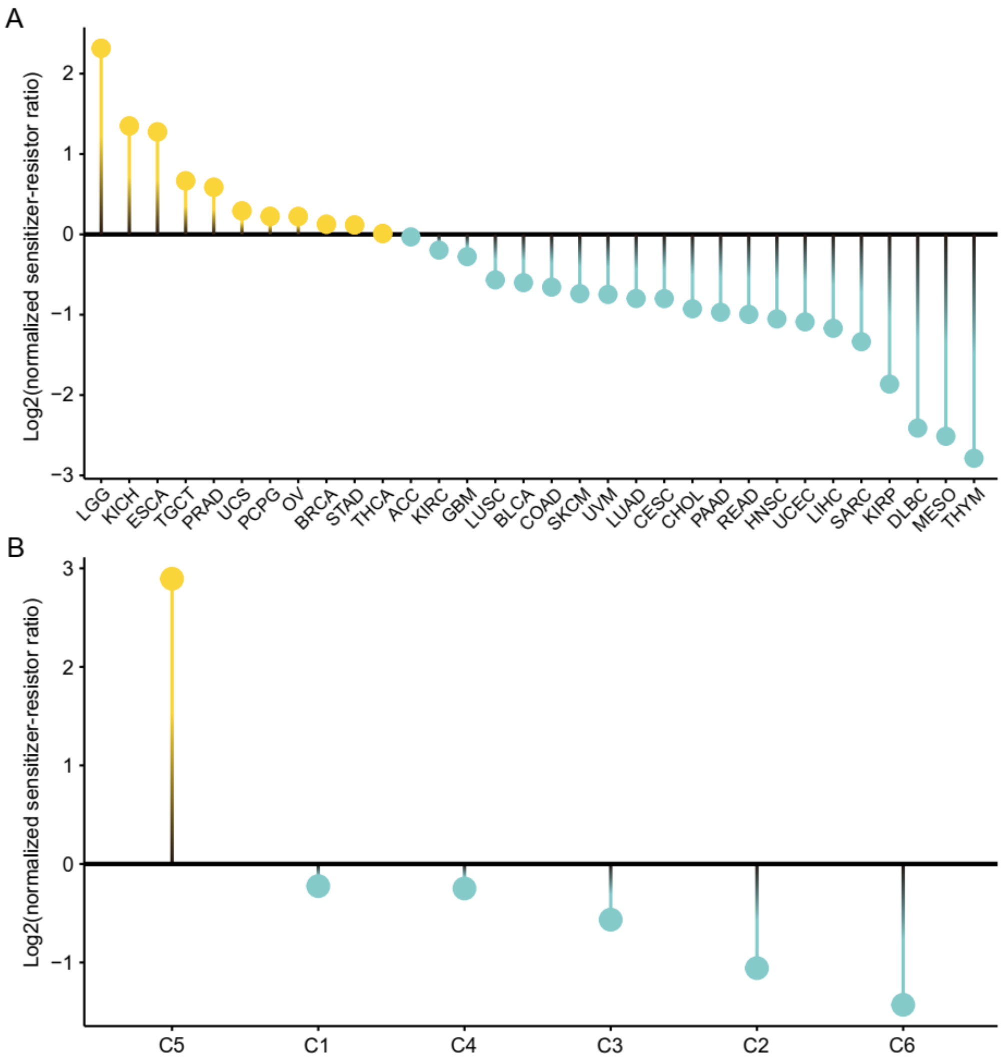

Figure S4. The ratio between the inactivation event numbers of sensitizers and resistors. (A) The distribution of normalized sensitizer-resistor ratio across different cancer types. (B) The distribution of normalized sensitizer-resistor ratio across different immune subtypes.

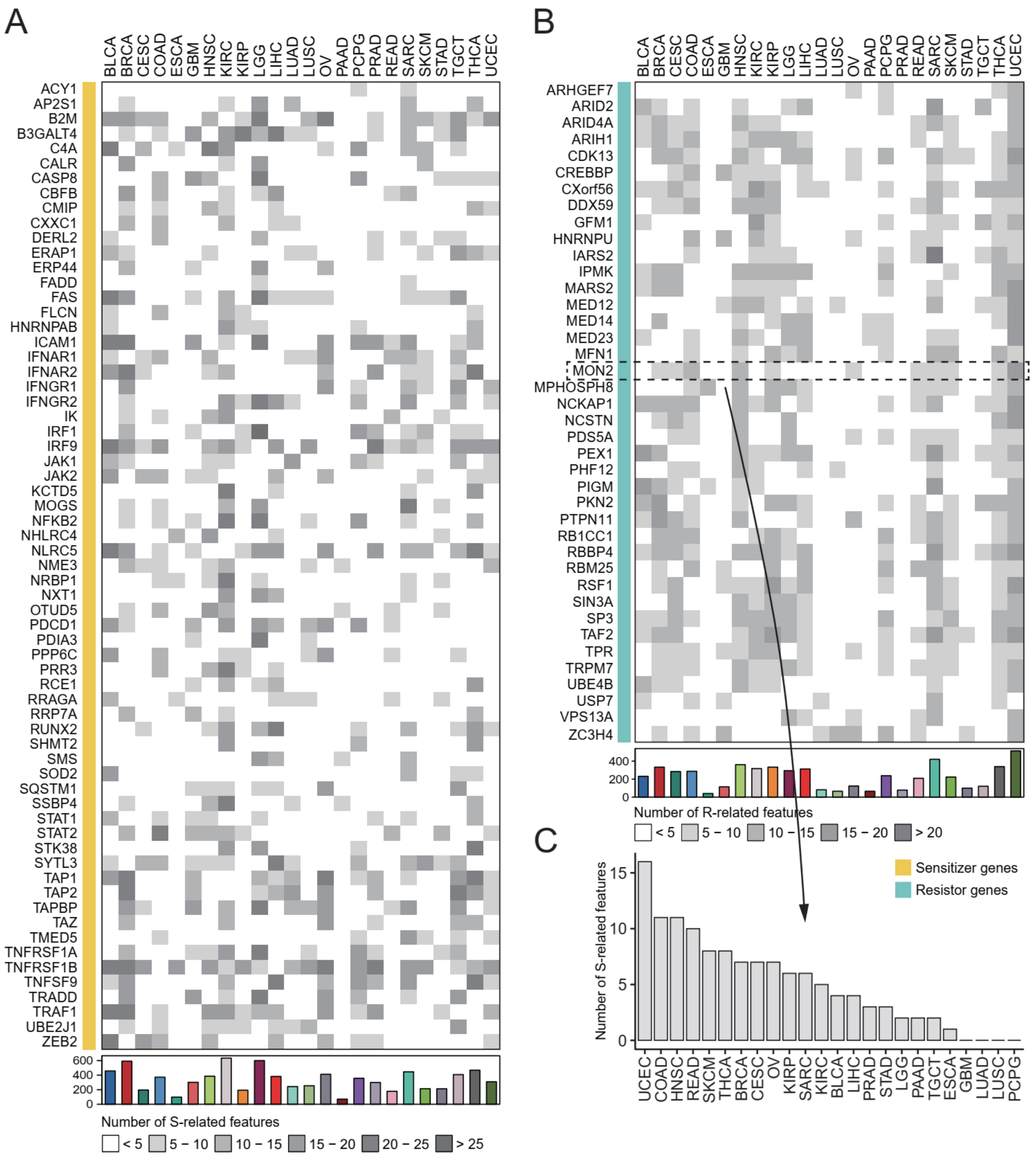

Figure S5. Functional characterization of sensitizers and resistors in each cancer type. (A) Presentation of the number of S-related features in each cancer type in each sensitizer gene. (B) Presentation of the number of R-related features in each cancer type in each resistor gene. (C) Distribution of S-related feature number of MON2 across different cancer types.

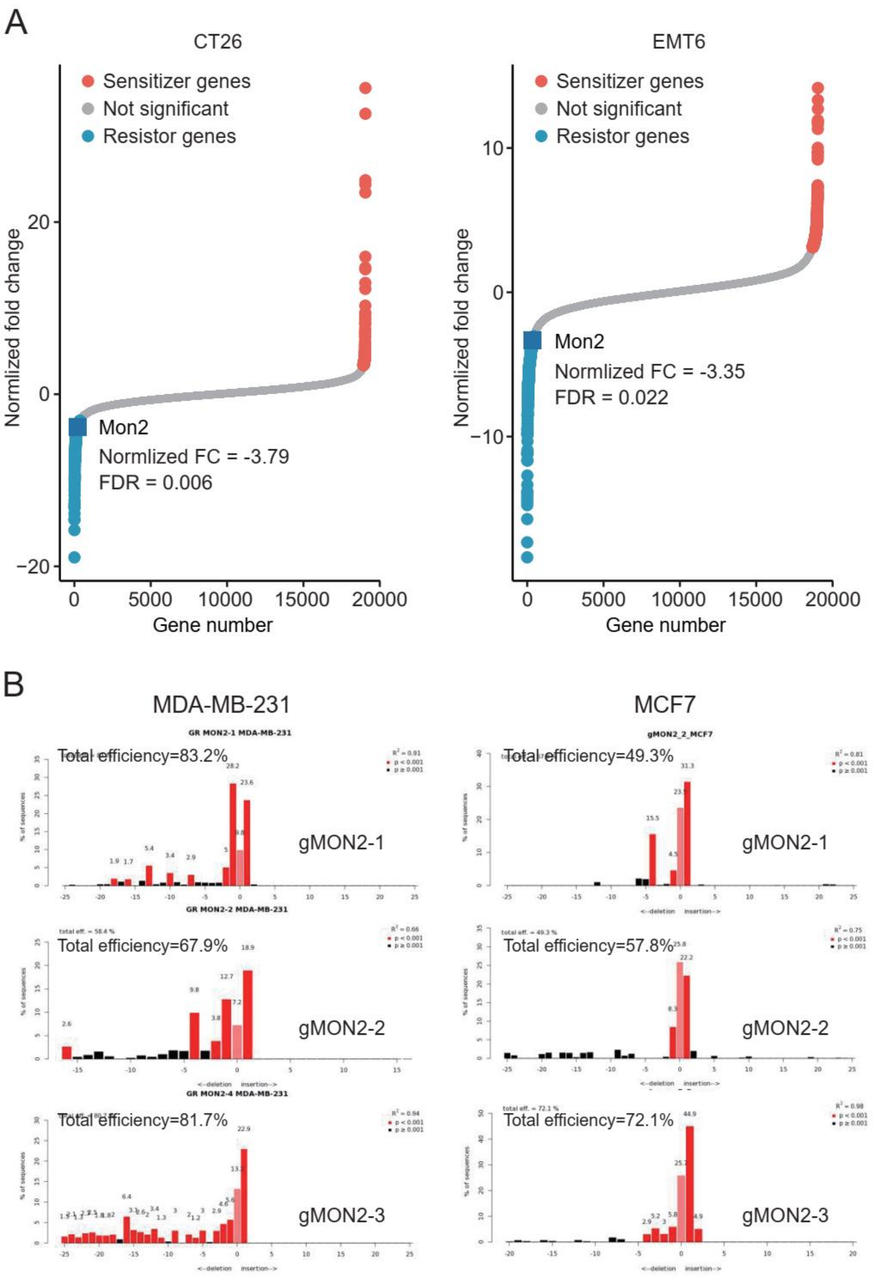

Figure S6. Experimental validation of the function of MON2. (A) Results of two screens which identified *MON2* as a significant resistor gene. (B) Determination of editing efficiencies of the MON2-targeting gRNAs.

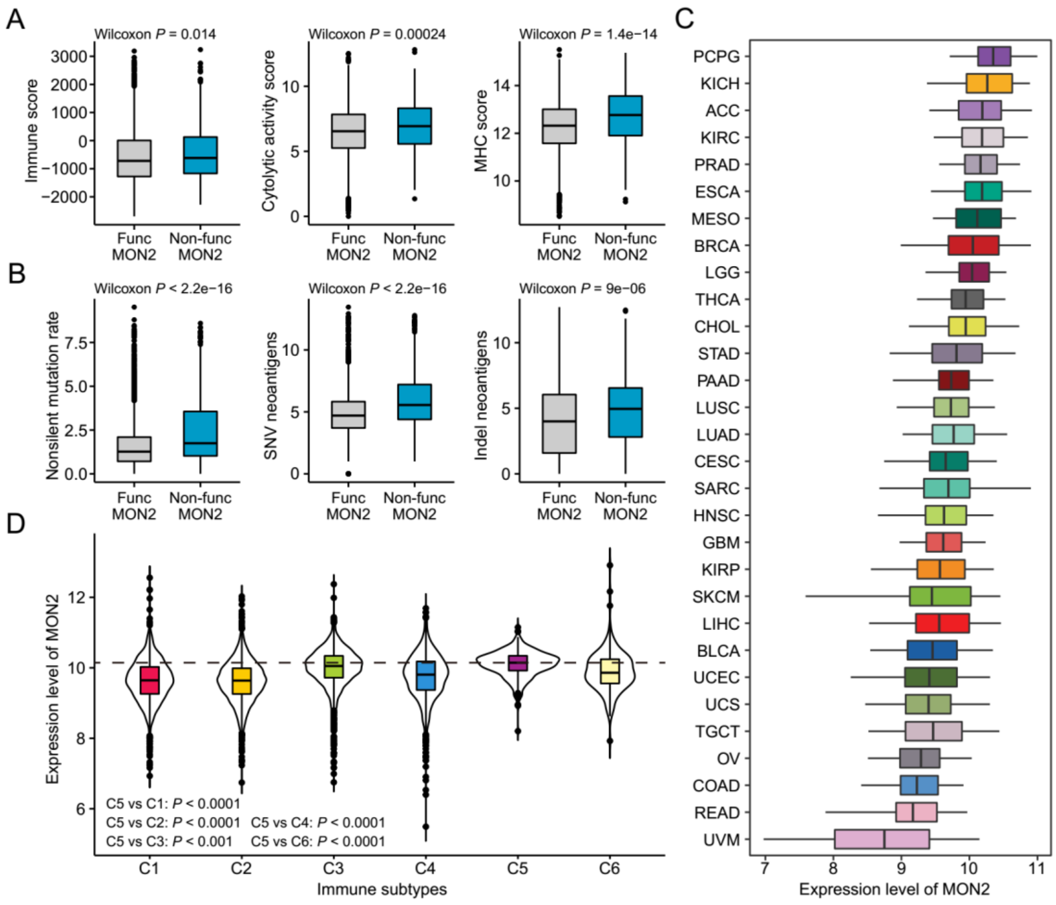

Figure S7. Overall characterization of MON2. (A) The association of MON2 with immune, cytolytic activity, and MHC scores. (B) The association of MON2 with mutational load, single nucleotide variant (SNV)-based neoantigen load, and insertion and deletion (indel)-based neoantigen load. (C) The distribution of MON2 expression across different cancer types. (D) The distribution of MON2 expression across different immune types. Statistical significance of difference was determined using Wilcoxon rank-sum test.

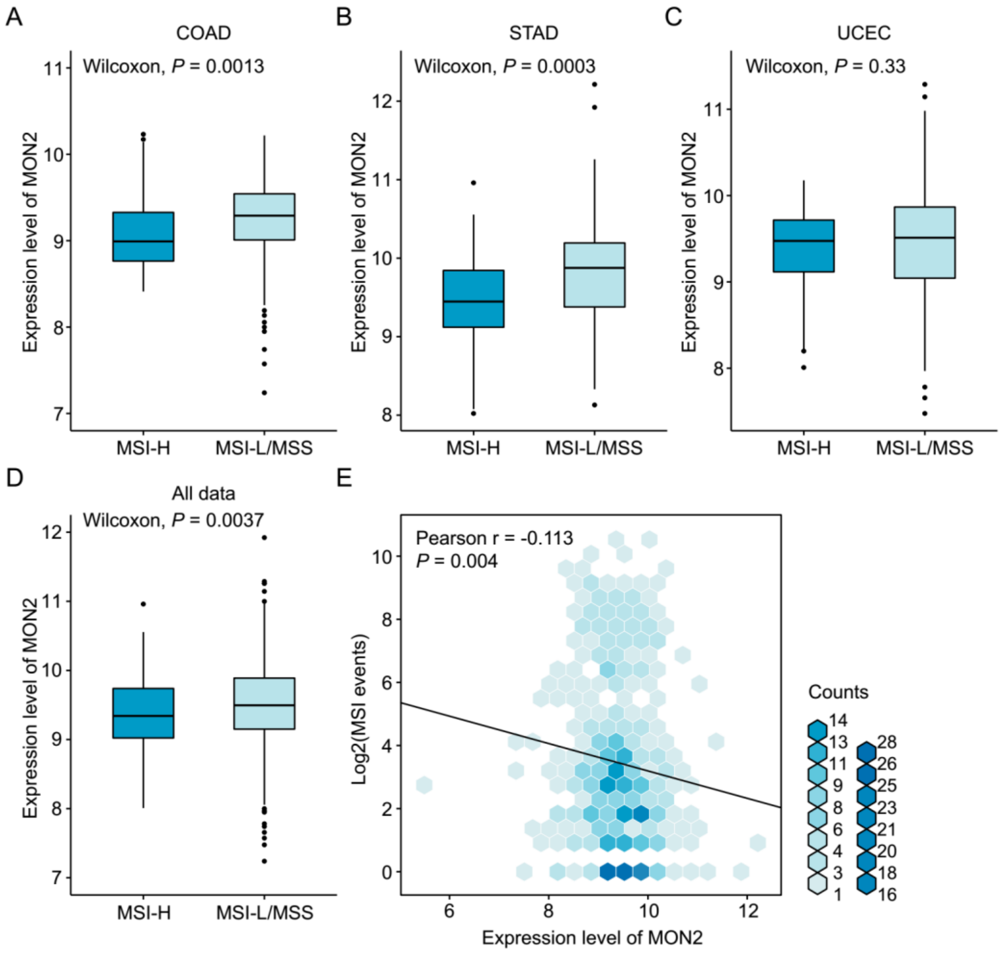

Figure S8. Relationship between MON2 and microsatellite instability (MSI). The difference of MON2 expression between MSI-H and MSI-L/MSS groups in COAD (A), STAD (B), UCEC (C), and all data (D) cohorts. Statistical significance of difference was determined using Wilcoxon rank-sum test. (E) Pearson correlation between MON2 expression and the number of MSI events.

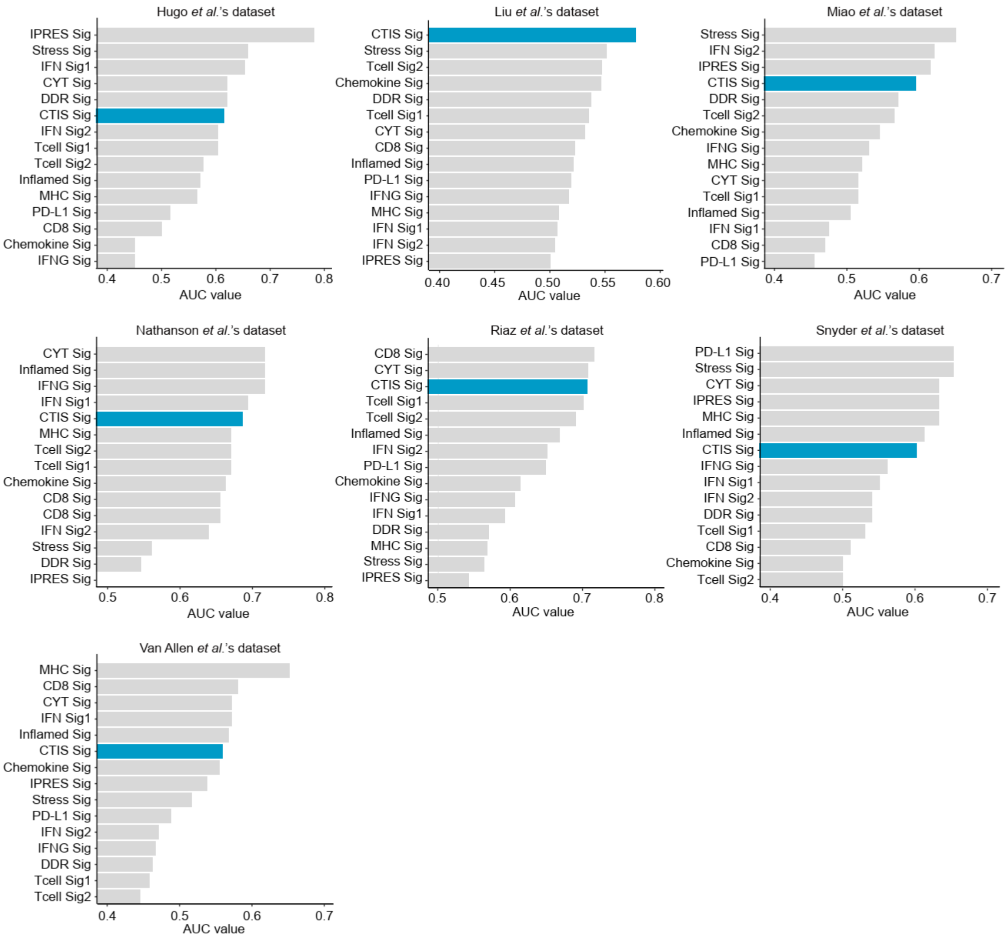

Figure S9. Comparison of the performance for predicting response to immunotherapy between CTIS and 14 published signatures in pretreatment datasets.

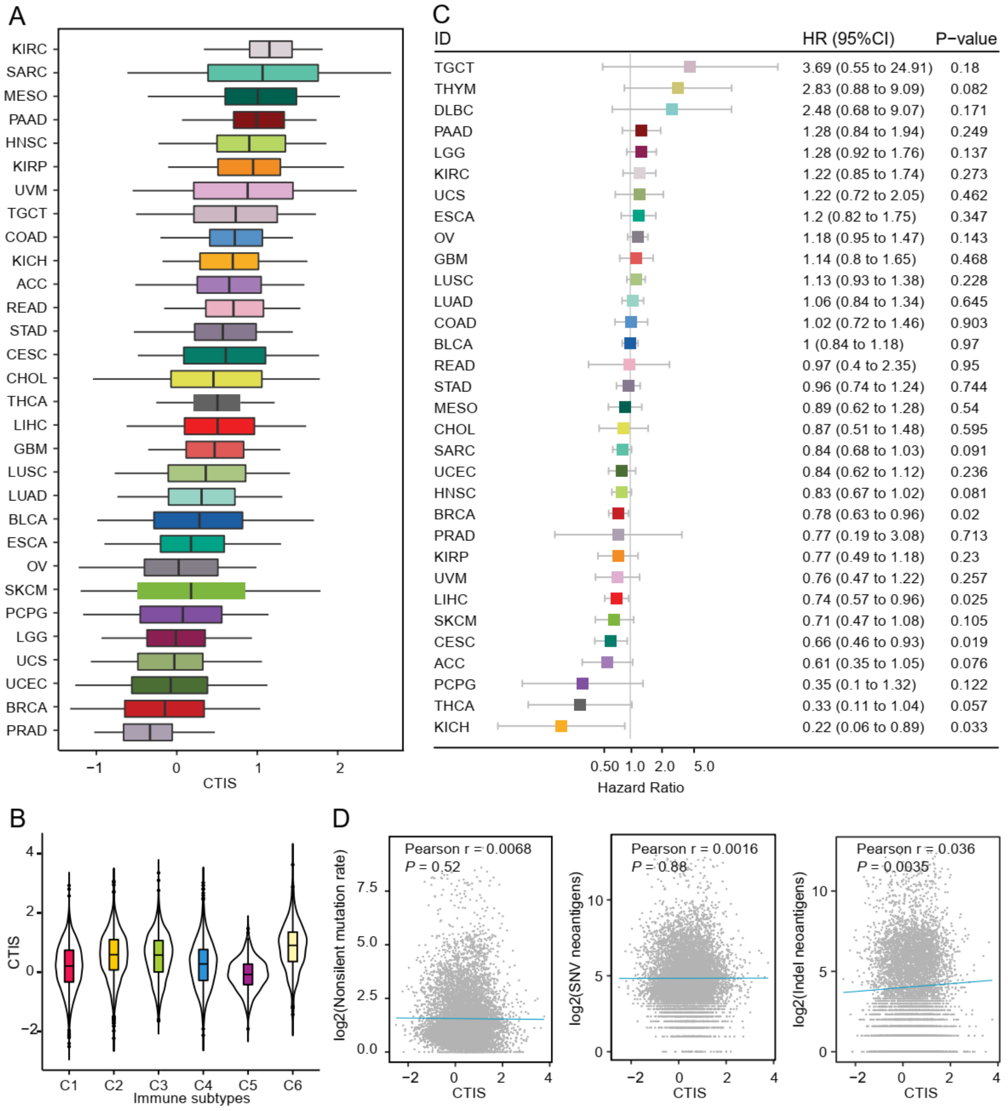

Figure S10. Characterization of CTIS in TCGA Pan-Cancer cohort. (A) Distribution of CTIS across different cancer types. (B) Distribution of CTIS across different immune subtypes. (C) Prognostic significance of CTIS across cancer types. (D) Pearson association of CTIS with mutational load, single nucleotide variant (SNV)-based neoantigen load, and insertion and deletion (indel)-based neoantigen load.

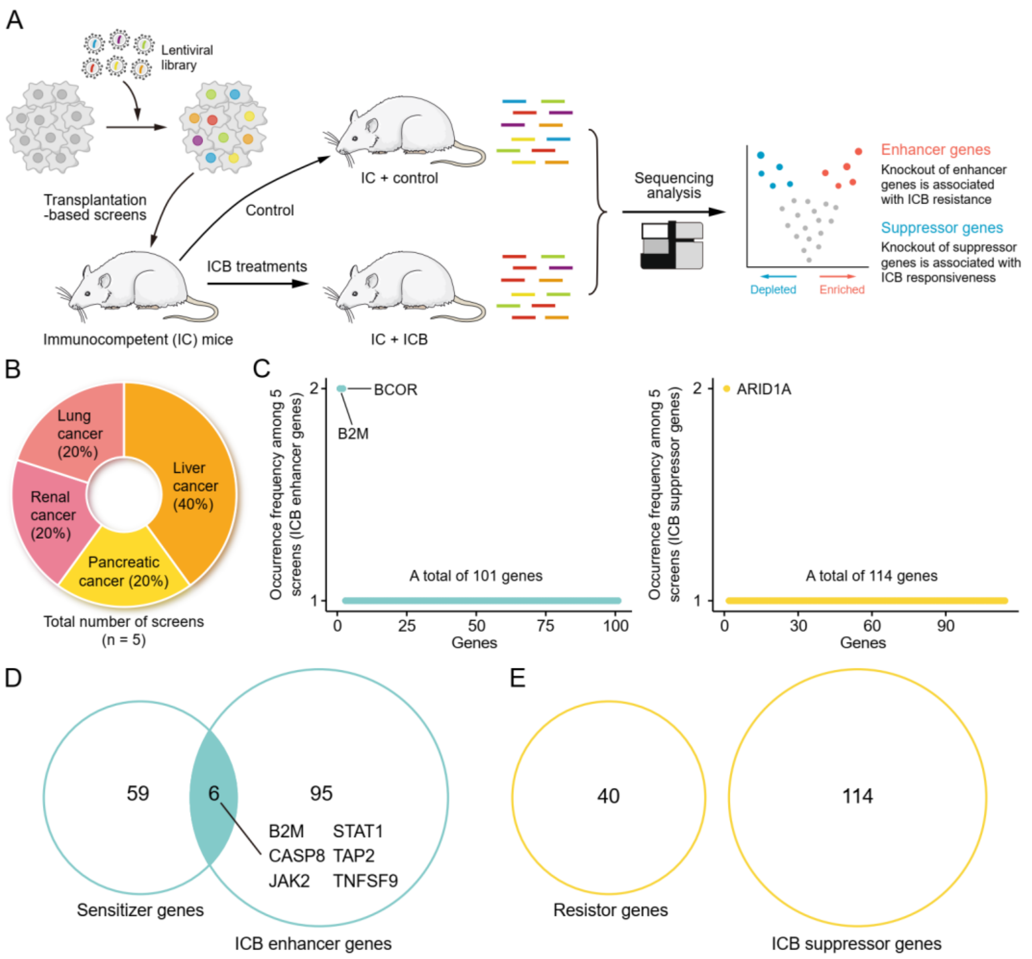

Figure S11. Summary of ICB-treated CRISPR screens. (A) Diagram summarizing the workflow of screens focused on identifying potential regulator genes mediating response to cancer immunotherapies. The definitions of enhancer genes and suppressor genes were also illustrated. (B) Presentation of cancer types studied by these screens. (C) Common enhancer genes (left) and suppressor genes (right) identified by different screens. (D) Intersection between sensitizer genes and ICB enhancer genes. (E) Intersection between resistor genes and ICB suppressor genes.
